# Saur and decline: Patterns in lizard imports to the US (2000–2022)

**DOI:** 10.1371/journal.pone.0333746

**Published:** 2025-10-22

**Authors:** Max Dolton Jones

**Affiliations:** Department of Fish and Wildlife Conservation, Virginia Tech, Blacksburg, Virginia, United States of America; HUN-REN Centre for Ecological Research, HUNGARY

## Abstract

The United States is an important component of global wildlife trade and benefits from the recording of trade data in the US Law Enforcement Management Information System (LEMIS). Despite its limitations, studies are beginning to highlight broad trends of US wildlife trade using this dataset which warrants further, more focused, investigations into taxon-specific data available within LEMIS data. I used LEMIS data to investigate patterns in lizard imports to the US between 2000 and 2022. Over 18.8 million whole lizards, comprised of 1,002 species, 259 genera, and 39 families, were imported to the US during this recording period. Similar to overall wildlife trade trends, many of the lizards were wild-sourced (61.7%) and likely imported due to the demands from the pet trade (99.8% for commercial purposes). The majority of the importations were of lizards from three families—*Gekkonidae*, *Agamidae*, and *Iguanidae*—which combined made up over 66% of all imports despite constituting only 7.7% of the family diversity. Overall, there was a decline in the number of lizard imports over time, yet there was an increase in the number of species being imported; with newly imported species increasing linearly. I highlight and discuss some of the patterns and implications that the lizard import data are suggesting, such as drivers of lizard imports, invasion risk, geographic collection “hotspots”, and limitations of the LEMIS data.

## 1. Introduction

There are several threats acting on species globally, contributing to the high rates of population declines observed across several taxa. These threats include habitat loss and degradation, climate change, direct mortality, invasive species, and a suite of other issues [1]. One major threat to many taxa is overexploitation for food, traditional medicine, the skin trade, and for the pet trade [2,3,4,5]. The United States in particular has been identified as a global hub for wildlife trade, and the US Fish and Wildlife Service’s Law Enforcement Management Information System (LEMIS) provides records for all imports and exports of plants and animals in the US [6,7,8]. This comprehensive dataset has recently been assessed, examining almost 3.5 million records of importations into the US, across a broad range of taxa constituting over 20,000 species (almost 30,000 when including CITES data; [8]). A staggering proportion of individuals were cleared for import into the US (99%), with many of the groups exhibiting high proportions of wild-sourced individuals [8]. However, the sustainability of wildlife trade was not directly assessed, and although harvesting individuals from wild populations can be achieved sustainably, monitoring of wild populations is often required [4,9].

There are an increasing number of studies investigating the underrepresented impacts of wildlife trade across taxa [10,7,11,8] and also studies focusing on reptiles specifically [12]. However, there is still a need for more targeted studies looking at smaller taxonomic units [13,14,15], particularly using the wealth of data available in the LEMIS dataset [6,16]. Despite recent assessments [8], it is important to focus in on groups of taxa more specifically so that we can identify if larger patterns of trade hold true, and if there are any unidentified threats or risks that become apparent during these deeper dives into taxon-level data [17]. Herein, I therefore investigate the LEMIS dataset focusing on lizard imports to the United States.

Lizards (order Squamata, suborder Sauria/Lacertilia) are a highly diverse and far-ranging group of reptiles, possessing almost 8,000 extant species with a rapid rate of species discovery and description [18,19]. Unsurprisingly, lizard populations are threatened globally due to several human-mediated factors [20,21]. Many lizard species are highly sought after for the pet trade [12], generally owed to the high level of diversity exhibited by lizard species, in regards to morphological traits, perceived or actual rarity, charismatic nature, and general attractiveness by potential pet owners as interesting display species [22]. Additionally, lizards are a highly diverse group with high rates of endemism [23,24,25]. Previous studies have demonstrated that collectors can target rare and unusual species, of which there are many lizard species, globally [22].

Even though there are known limitations to the LEMIS data, such as species misidentification, and general inaccuracies [26,27], this has been shown to be a valuable resource for investigating spatiotemporal trends of animal trade when little other data exists, and other formal trade recording mechanisms (i.e., CITES) are known to miss many species (especially reptiles; [28]). I present a focused evaluation of the imported lizards to the United States between 2000 and 2022, using data available from [8]. I demonstrate the main trends across all lizard imports, and further focus on the three most imported lizard families: *Gekkonidae*, *Agamidae*, and *Iguanidae*. I further provide summaries of major ports of entry for lizards into the US, and also highlight the areas of origin for these species and discuss the implications that these geographic biases may have.

## 2. Methods

I attained LEMIS data from [8] which contains US trade data between 2000 and 2022. I created a list of all lizard genera by comparing to family-level information available on The Reptile Database [19], and used this to subset the data to include lizards only. I caution additional studies using the “LIZARD” value of the “generic_name” column since an initial filtering proved to miss more than 80% of the total lizard imports. [8] provides a comprehensively cleaned and summarised version of the LEMIS dataset where scientific names were unified by conforming to recent and relevant taxonomic authorities. The quantity values within the LEMIS data can prove to be unreliable and inaccurate, and thus I opted to use count data only (unit = “NO” [number]) which represents a whole individual, both dead or alive. I further limited data review to records containing the description code “LIV” for live individuals only. By applying a filter for live individuals, this only removed 771 individuals across 47 species, 28 genera, and 11 families (S1 Table). I removed any NA’s still present in the “quantity” data column (thus removing the data entry). Although I include all remaining records within the data summaries, I removed any unconfirmed species when calculating the number of species represented in the data. Due to the scale of the LEMIS data, it is difficult to identify all errors without having a focused look at taxonomic subsets in the data (and taxonomic expertise or experience). During my review of the lizard subset, I noticed four naming errors where generic names did not meet currently accepted naming conventions. I therefore corrected *Lacerta vivipara* to *Zootoca vivipara*, *Cordylus giganteus* to *Smaug giganteus*, and *Hypsilurus boydii* and *H. dilophus* to the genus *Lophosaurus* (constituting 34 entries and 233 lizards). Although I could not identify any other naming errors, it is likely that other errors are still present.

Eskew et al. [7] highlight a number of cautions with interpreting some of the data columns within the LEMIS data; namely, the “country_imp_exp” which reports the most recent country that import was located, and “country_origin” which is the original location (point of origin) of the animals. However, I summarised the country of origin data by separating results into individuals sourced from the wild, and individuals sourced from captivity. Several records reported the country of origin as the US, which likely represent data-entry errors because it is unlikely that records being imported into the US originated from the US. I therefore removed any records that recorded the US as the country of origin, which removed 17,716 lizards from the dataset (S2 Table). I created Sankey diagrams to demonstrate the number of imports across all genera, countries and ports of entry for *Agamidae*, *Gekkonidae*, and *Iguanidae*. Then, to create “hotspot” maps of countries where future assessments of harvesting sustainability may be warranted, I filtered the import data to include only wild-caught records and further grouped data by genus and country of origin.

I used R v.4.4.0 [29] and RStudio v.2024.4.1.748 30] to review, manipulate, and summarize data. I used *readr* v.2.1.5 [31] to read in data, *dplyr* v.1.1.4 [32] for filtering and manipulating data, *ggplot2* v.3.5.1 [33], *scico* v.1.5.0 [Pedersen and Crameri 2023] [34], *ti*dyterra** v.0.6.0 [35], and *ggsankey* v.0.0.99999 [36] for visualizing import data, and *terra* v.1.7–71 [37] for reading-in and working with spatial datasets. I performed final figure edits using Inkscape v.1.2.1.

## 3. Results

### 3.1 Trends across all lizard imports

Between 2^nd^ January 2000 and 30^th^ June 2022, there were 18,803,181 live whole lizards imported to the United States. This was comprised of 1,002 species across 259 genera, and 39 families. Lizards were imported to 49 ports of entry in 26 States. Most imports were cleared for entry (99.8%), and constituted primarily wild-sourced individuals (61.7%). The second largest source of imports was from individuals that were bred in captivity (34.2%); whereas individuals born into captivity only made up 0.2% of imports. Some individuals were sourced from ranching operations (3.3%), and 0.6% of all imports came from unknown sources. There were very few imports (<0.0001%) that were commercially bred or from confiscations. The purpose of importations was overwhelmingly for commercial purposes (99.8%), presumably for the pet trade. Though there were some imports (<0.1%) across several other categories (hunting trophies, personal, scientific, breeding in captivity, zoo, education, and for circuses; in descending order of proportion).

**Fig 1:**
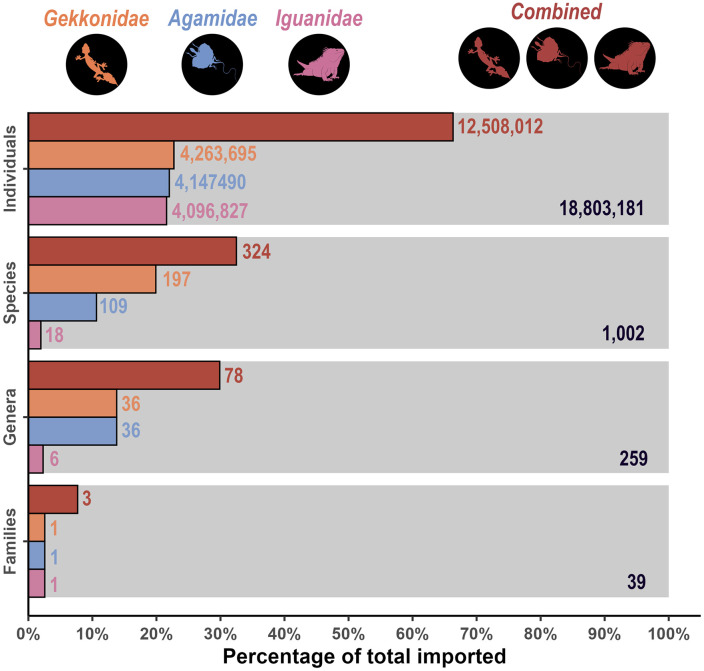
Summary of all lizard imports to the Unites States within the US Fish and Wildlife Service’s Law Enforcement Management Information System (LEMIS) dataset between 2000-2022. Results across all imports are displayed in grey (100%), and *Gekkonidae*, *Agamidae*, *Iguanidae*, and a combination of all three lizard families are represented by orange, blue, pink, and red, respectively. Numbers on the right of each bar represent the imported number (i.e., number of imported individuals, species, genera, or families). Numbers on the bottom right of the grey bars show the total number imported across each category.

The most imported genus of lizard across all lizard imports was *Iguana* (21.5%), followed by *Hemidactylus* (13.3%), *Pogona* (8.2%), *Physignathus* (7.7%), and *Takydromus* (7.5%). The most imported species (from individuals identified to the species level) across all imports were of *Iguana iguana* (green iguana; 21.5%) which was followed by *Pogona vitticeps* (bearded dragon; 8.2%), *Physignathus cocincinus* (Indochinese water dragon; 7.6%), *Takydromus sexlineatus* (southern grass lizard; 6.3%) and *Hemidactylus frenatus* (common house gecko; 3%). The majority of imports occurred across three families: *Agamidae*, *Gekkonidae*, and *Iguanidae* (Fig 1). A combination of these three families – which only constituted 7.7% of the family diversity – comprised 66.5% of all imports, 32.3% of the species diversity, and 30.1% of the imported genera. This suggests that select species from these families are most commonly imported, presumably due to the preference of hobbyists and other consumers. Additionally, these three families were imported to 91.3% of the ports of entry into the US, and 88.5% of the import States.

Wild-sourced lizards were primarily imported from Vietnam (45.4%), followed by the United Republic of Tanzania (8.1%; imported between 2000–2016 prior to the export ban), Indonesia (8.1%), Egypt (5.5%) and Togo (4.4%). Lizards sourced from captivity (born and bred) were primarily imported from El Salvador (59.2%), Colombia (16.8%), Nicaragua (8.1%), Thailand (5.3%), and Vietnam (2.8%). Imports (across all sources) primarily occurred into Miami, Florida (55.7%) and Los Angeles, California (38.7%), and a relatively high number into Dallas/Fort Worth, Texas (2.8%). All other ports of entry had less than 1% of imports occur there.

### 3.2 Gekkonidae

**Fig 2:**
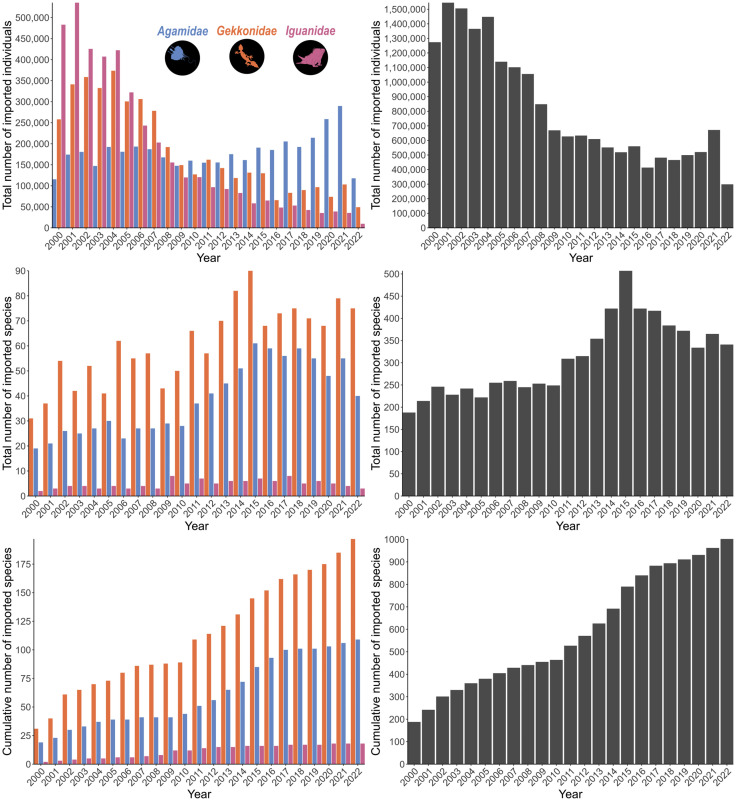
Patterns of imported lizards according to the United States Fish and Wildlife Service’s Law Enforcement Management Information System (LEMIS) dataset between 2000-2022. Grey bars (right charts) represent trends for all imported lizard species, orange, blue, and pink depict lizards in the family *Gekkonidae*, *Agamidae*, and *Iguanidae*, respectively (left). Top-left: number of imported lizards over time for the three most imported families, center-left: number of species reported each year for the three most imported families, bottom-left: cumulative number of species reported over time for the three most imported families, top-right: number of imported lizards over time for all lizard imports, center-right: number of species reported each year for all lizard imports, bottom-right: cumulative number of species reported over time for all lizard imports.

**Fig 3:**
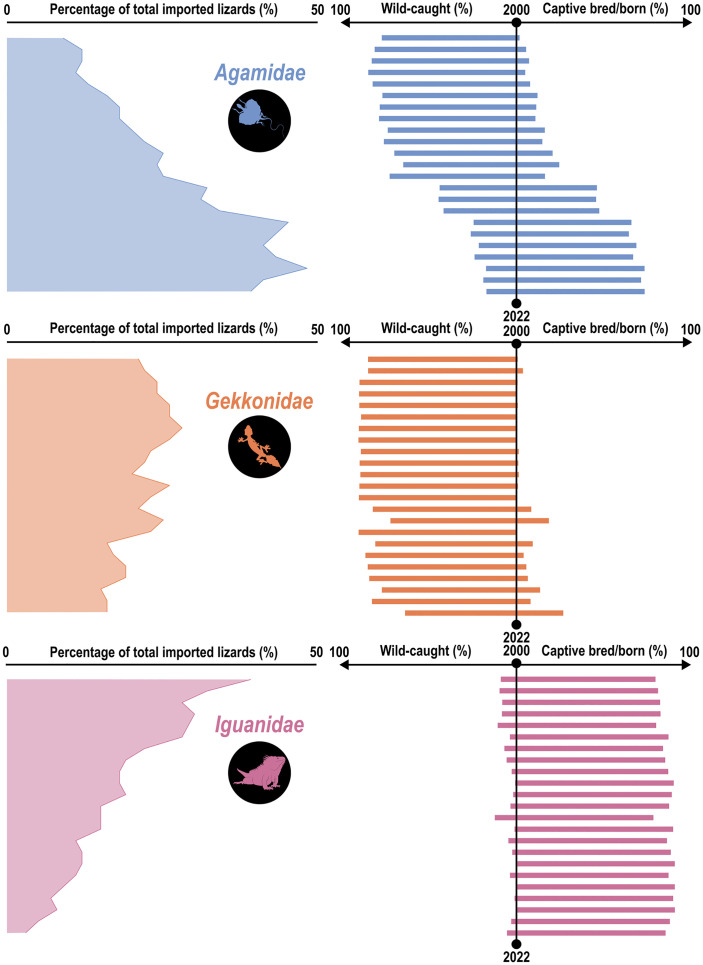
Patterns of imported lizards according to the United States Fish and Wildlife Service’s Law Enforcement Management Information System (LEMIS) dataset, between 2000-2022. Blue, orange, and pink plots depict lizards from the *Agamidae*, *Gekkonidae*, and *Iguanidae* family, respectively. Left plots depict the percentage of imported lizards that were comprised of individuals from each of the highlighted families across the reporting period (top to bottom of each plot). Right plotsshow the percentage of imported lizards from each family that was comprised of individuals sourced from the wild or from captivity.

There were 4,263,695 live whole gekkonid lizards imported to the United States during the recording period, which constituted 197 species (19.7%) and 36 genera (13.9%). Gekkonids were imported to 28 ports of entry (60.9%) in 19 States (73.1%). Gekkonid imports were overwhelmingly sourced from the wild (96.2%), with only 3% from captive-bred populations. The number of imported gekkonids decreased sharply throughout the recording period, but the number of unique species, and species imported for the very first time increased over time (Fig 2). Additionally, the proportion of imports made up of gekkonids remained relatively constant (and high) throughout the importation period (Fig 3). The source of imports was consistently biased towards wild-sourced individuals, although there were more captive bred/born individuals later in the dataset (Fig 3).

The most imported genus of gekkonid lizards was by far *Hemidactylus* (58.8%), which was followed by another highly-imported genus, *Gekko* (19.6%). Three other genera in the top-five most traded gekkonids include *Lygodactylus* (3.5%), *Phelsuma* (3.4%), and *Gehyra* (3.1%). The most imported gekkonid species were of *Hemidactylus frenatus* (common house gecko; 13%), *Gekko gekko* (tokay gecko; 9.2%), *Ptychozoon kuhli* (Kuhl’s parachute gecko; 2.9%), *Gehyra mutilata* (stump-tailed gecko; 2.8%) and *Gekko vittatus* (lined gecko; 2.6%). Wild-sourced gekkonids were primarily imported from Vietnam (57.7%), followed by Indonesia (15.4%), Thailand (6.4%), the United Republic of Tanzania (6.3%), and Madagascar (3.7%). Geckos that were born or bred in captivity originated from Vietnam (57.5%), Thailand (20.7%), Indonesia (3%), Germany (3%), and Venezuela (2.3%). Gekkonid imports (across all sources) primarily occurred into Los Angeles, California (54.7%), Miami, Florida (37.3%), and Dallas/Fort Worth, Texas (6.3%). All other ports of entry had less than 1% of gekkonid imports occur there.

**Table 1:** Source of importation into the United States for all imported genera in *Gekkonidae*, *Agamidae*, and *Iguanidae* according to the United States Fish and Wildlife Service’s Law Enforcement Management Information System (LEMIS) dataset between 2000-2022.

Genus	Source	Imported lizards	Percentage of genus (%)
*Agamidae*			
*Acanthocercus*	Captive-bred	9	5.7
	Wild	150	94.3
*Acanthosaura*	Captive-bred	9669	4.1
	Captive-born	100	0.0
	Ranching	100	0.0
	Unknown	1708	0.7
	Wild	222841	95.1
*Agama*	Captive-bred	3373	1.5
	Ranching	15100	6.7
	Unknown	4883	2.2
	Wild	201199	89.0
*Amphibolurus*	Captive-bred	16	84.2
	Captive-born	1	5.3
	Unknown	1	5.3
	Wild	1	5.3
*Aphaniotis*	Wild	40	100.0
*Bronchocela*	Captive-bred	11	0.4
	Wild	2574	99.6
*Calotes*	Captive-bred	1987	3.5
	Ranching	200	0.4
	Unknown	1450	2.6
	Wild	52972	93.6
*Ceratophora*	Captive-bred	85	80.2
	Wild	21	19.8
*Chlamydosaurus*	Captive-bred	22352	57.8
	Captive-born	1278	3.3
	Wild	15052	38.9
*Cophotis*	Captive-bred	17	63.0
	Wild	10	37.0
*Ctenophorus*	Captive-bred	48	70.6
	Captive-born	4	5.9
	Wild	16	23.5
*Draco*	Captive-bred	419	2.3
	Captive-born	50	0.3
	Unknown	129	0.7
	Wild	17630	96.7
*Gonocephalus*	Captive-bred	316	2.0
	Unknown	31	0.2
	Wild	14962	97.0
*Hydrosaurus*	Captive-bred	2592	20.1
	Captive-born	1487	11.6
	Wild	8770	68.3
*Hypsilurus*	Captive-bred	61	8.2
	Wild	684	91.8
*Intellagama*	Captive-bred	229	75.8
	Captive-born	20	6.6
	Wild	53	17.5
*Japalura*	Captive-bred	19647	11.9
	Captive-born	1	0.0
	Ranching	300	0.2
	Unknown	70	0.0
	Wild	144480	87.8
*Laudakia*	Captive-bred	414	3.6
	Wild	11216	96.4
*Leiolepis*	Captive-bred	2503	5.5
	Ranching	100	0.2
	Unknown	86	0.2
	Wild	43090	94.1
*Lophognathus*	Captive-bred	57	1.2
	Wild	4641	98.8
*Lophosaurus*	Captive-bred	46	85.2
	Wild	8	14.8
*Lyriocephalus*	Captive-bred	20	90.9
	Wild	2	9.1
*Malayodracon*	Wild	12	100.0
*Otocryptis*	Captive-bred	3	100.0
*Phrynocephalus*	Captive-bred	1177	10.2
	Wild	10383	89.8
*Physignathus*	Captive-bred	52608	3.7
	Captive-born	452	0.0
	Ranching	512	0.0
	Unknown	13342	0.9
	Wild	1371779	95.3
*Pogona*	Captive-bred	1514603	98.2
	Captive-born	838	0.1
	Ranching	6	0.0
	Unknown	26	0.0
	Wild	26637	1.7
*Psammophilus*	Captive-bred	12	100.0
*Pseudocalotes*	Wild	51	100.0
*Pseudotrapelus*	Captive-bred	200	15.2
*Pseudotrapelus*	Wild	1117	84.8
*Saara*	Captive-bred	760	62.7
	Captive-born	100	8.2
	Wild	353	29.1
*Salea*	Captive-bred	12	100.0
*Trapelus*	Wild	5688	100.0
*Tympanocryptis*	Captive-bred	87	83.7
	Wild	17	16.3
*Uromastyx*	Captive-bred	25592	8.3
	Captive-born	7256	2.4
	Ranching	3556	1.2
	Wild	270772	88.0
*Xenagama*	Captive-bred	433	7.4
	Wild	5454	92.6
*Gekkonidae*			
*Afroedura*	Captive-bred	24	92.3
	Wild	2	7.7
*Agamura*	Captive-bred	205	99.0
	Wild	2	1.0
*Ailuronyx*	Captive-bred	29	64.4
	Wild	16	35.6
*Alsophylax*	Captive-bred	2	20.0
	Wild	8	80.0
*Blaesodactylus*	Captive-bred	16	0.5
	Wild	2922	99.5
*Bunopus*	Captive-bred	14	0.9
	Wild	1585	99.1
*Chondrodactylus*	Captive-bred	132	34.7
	Captive-born	1	0.3
	Wild	247	65
*Christinus*	Captive-bred	1	0.1
	Captive-born	2	0.1
	Wild	1557	99.8
*Cnemaspis*	Captive-bred	31	1.6
	Wild	1936	98.4
*Cyrtodactylus*	Captive-bred	780	3.5
	Unknown	179	0.8
	Wild	21054	95.6
*Cyrtopodion*	Captive-bred	64	0.4
	Captive-born	2	0.0
	Wild	15297	99.6
*Dixonius*	Captive-bred	8	25
	Wild	24	75
*Ebenavia*	Captive-bred	23	0.9
	Wild	2629	99.1
*Geckolepis*	Captive-bred	258	1.8
	Unknown	300	2.0
	Wild	14141	96.2
*Gehyra*	Captive-bred	561	0.4
	Captive-born	7	0.0
	Wild	131928	99.6
*Gekko*	Captive-bred	18694	2.2
	Captive-born	690	0.1
	Ranching	300	0.0
	Unknown	7286	0.9
	Wild	807970	96.6
*Hemidactylus*	Captive-bred	75505	3
	Captive-born	1120	0.0
	Ranching	9606	0.4
	Unknown	5173	0.2
	Wild	2414644	96.3
*Hemiphyllodactylus*	Captive-bred	41	10.4
	Wild	352	89.6
*Heteronotia*	Captive-bred	19	100.0
*Homopholis*	Captive-bred	104	1.4
	Ranching	20	0.3
	Unknown	1488	20.2
	Wild	5742	78.1
*Lepidodactylus*	Captive-bred	756	84.3
	Captive-born	1	0.1
	Wild	140	15.6
*Lygodactylus*	Captive-bred	2168	1.5
	Captive-born	112	0.1
	Ranching	460	0.3
	Unknown	100	0.1
	Wild	144379	98.1
*Matoatoa*	Wild	782	100.0
*Mediodactylus*	Captive-bred	31	100.0
*Nactus*	Captive-bred	26	13.8
	Wild	162	86.2
*Pachydactylus*	Captive-bred	1537	1.6
	Unknown	1440	1.5
	Wild	93070	96.9
*Paroedura*	Captive-bred	630	1.7
	Ranching	40	0.1
	Unknown	158	0.4
	Wild	36187	97.8
*Phelsuma*	Captive-bred	23193	15.9
	Captive-born	10	0.0
	Ranching	1055	0.7
	Unknown	2	0.0
	Wild	121494	83.4
*Pseudogekko*	Captive-bred	70	92.1
	Wild	6	7.9
*Ptenopus*	Captive-bred	19	73.1
	Wild	7	26.9
*Ptychozoon*	Captive-bred	406	0.3
	Captive-born	20	0.0
	Wild	127069	99.6
*Stenodactylus*	Captive-bred	1117	1.2
	Unknown	2455	2.6
	Wild	92279	96.3
*Tenuidactylus*	Captive-bred	9	15.3
	Wild	50	54.7
*Trachydactylus*	Captive-bred	9	100.0
*Tropiocolotes*	Captive-bred	251	2.1
	Wild	11502	97.9
*Uroplatus*	Captive-bred	1672	3.1
	Captive-born	55	0.1
	Ranching	402	0.7
	Unknown	251	0.5
	Wild	52041	95.6
*Iguanidae*			
*Conolophus*	Wild	262	100
*Ctenosaura*	Captive-bred	35021	68.6
	Captive-born	19	0.0
	Unknown	367	0.7
	Wild	15570	30.5
*Cyclura*	Captive-bred	10	100
*Dipsosaurus*	Captive-bred	12	7.5
*Iguana*	Captive-bred	3731298	92.2
	Captive-born	18	0.0
	Confiscated or seized	1	0.0
	Ranching	5917	0.1
	Unknown	10274	0.3
	Wild	297988	7.4
*Sauromalus*	Captive-bred	15	83.3
	Wild	3	16.7

*Captive-bred*: the successful breeding of lizards in captivity resulting in the birth/hatching of imported lizards; *captive-born*: imported individuals were born in captivity due to the importation of a gravid female lizard; *wild*: individuals were harvested from wild populations; *ranching*: imported lizards were harvested from the wild as eggs or juveniles and subsequently raised in captivity; *unknown*: source is unknown; *confiscated or seized*: imports were a result of a confiscation or seizure by law enforcement.

### 3.3 Agamidae

There were 4,147,490 live whole agamid lizards imported to the United States during the recording period, which constituted 109 species (10.9%) and 36 genera (13.9%). Agamids were imported to 33 ports of entry (71.7%) in 23 States (88.5%). Agamid imports were primarily sourced from the wild (58.7%), yet a relatively high number were sourced from captive-breeding (40%). In contrast to the pattern across all lizard imports, the number of imported agamids generally increased over time (except a decline between 2021 and 2022). Additionally, the number of unique species, and species imported for the very first time increased over time (Fig 2). The proportion of imports made up of agamids increased substantially over time (Fig 3), with a similarly-patterned shift from wild-caught individuals to individuals sourced from captive-breeding (Fig 3).

The most imported genus of agamid lizards was *Pogona* (37.2%), which was followed closely by *Physignathus* (34.7%), and then by *Uromastyx* (7.4%), *Acanthosaura* (5.7%), and *Agama* (5.5%). The most imported agamid species were of *Pogona vitticeps* (bearded dragon; 37%), *Physignathus cocincinus* (Indochinese water dragon; 34.6%), *Agama agama* (rainbow lizard; 3.6%), *Uromastyx dispar* (Sudan mastigure; 3.1%) and *Uromastyx geyri* (Sahara mastigure; 3.1%). Wild-sourced agamids were primarily imported from Vietnam (68.9%), followed by Mali (7.4%), China (5.5%), Togo (2.5%), and Egypt (2.3%). Agamids sourced from captivity were exported from El Salvador (70.7%) primarily, and also Thailand (17.5%), Vietnam (3.8%), Indonesia (1.6%) and China (1.2%). Agamid imports (across all sources) primarily occurred into Miami, Florida (59.2%), Los Angeles, California (35.3%), Dallas/Fort Worth, Texas (1.8%), Seattle, Washington (1.2%), and Chicago, Illinois (1.1%). All other ports of entry had less than 1% of agamid imports occur there.

### 3.4 Iguanidae

There were 4,096,827 live whole iguanid lizards imported to the United States during the recording period, which constituted 18 species (1.8%) and 6 genera (2.3%). Iguanids were imported to 30 ports of entry (65.2%) in 16 States (61.5%). Iguanid imports were primarily sourced from captive-breeding (91.9%), and unlike the general lizard imports and the other most-imported families (*Gekkonidae* and *Agamidae*), very few individuals were sourced from the wild (7.7%). Congruent with the general import data, the number of imported iguanids decreased over time. Additionally, the number of unique species, and species imported for the very first time remained relatively constant across the dataset (Fig 2). The proportion of imports made up of iguanids decreased substantially over time (Fig 3), with a consistently low proportion of individuals being wild-caught (Fig 3).

The most imported genus of iguanid lizards was overwhelmingly *Iguana* (98.7%), which was followed by *Ctenosaura* (1.3%), *Conolophus* (0.006%), *Sauromalus* (0.004%), and *Dipsosaurus* (0.003%). The most imported iguanid species were of *Iguana iguana* (green iguana; 98.6%), and the other top-five species were all from the *Ctenosaura* genus: *C. quinquecarinata* (five-keeled spiny-tailed iguana; 0.7%), *C. similis* (common spiny-tailed iguana; 0.5%), *C. pectinata* (western spiny-tailed iguana; 0.02%)*,* and *C. palearis* (Motagua spiny-tailed iguana; 0.006%). Wild-sourced iguanids were primarily imported from El Salvador (44.4%), followed by Suriname (27.9%), Colombia (21.2%), Honduras (3.8%), and Guatemala (1%). Captive bred/born individuals were imported from from El Salvador (70%), Colombia (27.9%), Nicaragua (1.1%), Guatemala (0.8%), and Liechtenstein (0.1%). Iguanid imports (across all sources) primarily occurred into Miami, Florida (78.8%), and Los Angeles, California (20.9%). All other ports of entry had less than 1% of iguanid imports occur there.

### 3.5 Combined summary

When summarising the imports across *Agamidae, Gekkonidae*, and *Iguanidae*, there were 12,508,012 (66.5%) live whole lizards imported to the United States during the recording period, which constituted 324 species (32.3%) and 78 genera (30.1%); yet only comprised 7.7% of the family diversity. These combined families were imported to 42 ports of entry (91.3%) in 23 States (88.5%). Collectively, the combined imports were primarily wild-caught individuals (54.7%), or sourced from captive-breeding (44.4%).

## 4. Discussion

### 4.1 Patterns of lizard importation

My investigation demonstrates the vast scale of lizard importation into the US between 2000 and 2022, where approximately 18.8 million lizards representing 1,002 species across 259 genera and 39 families, have been imported to the US. These importations have been primarily for commercial purposes (99.8%), likely driven by demand from the pet trade. There was considerable bias in the dataset where the majority of imports (66.5%) were contributed by three families primarily: *Gekkonidae*, *Agamidae*, and *Iguanidae*; despite these only representing 7.7% of the family diversity. After 2012/2013, there was a spike in the number of species being imported across the entire dataset, and the cumulative number of species also exhibited a positive linear pattern (Fig 3). Thus, not only were the number of species being imported very high during this period, there were several new species that had not been imported to the US previously (within the LEMIS dataset) now being imported. However, during this time, the number of lizards being imported were at the lowest reported across the entire recording period. These patterns may be partly driven by updated reporting requirements by USFWS, particularly for spikes in species’ numbers which is predicated on a port agents’ ability to accurately identify lizards [26]. This is a general pattern across all lizard imports, but may not be present across all families. I summarized the top three imported families and demonstrated that *Gekkonidae* also follow this pattern (the most traded lizard family within the LEMIS dataset), but this does not appear to completely hold true for agamids or iguanids (Fig 2). For iguanids specifically, the number of species remains fairly constant across the recording period, and agamids actually show an increase in imported lizards over time (except for 2021–2022).

These patterns may be particularly acute for lizards due to being the most diverse reptile taxa with almost 8,000 extant species and a rapid rate of species descriptions. For example, there was an increase from ~5,800 species in 2013 to ~7,300 species in 2022 [19]. Many of these new species are likely due to the splitting of species complexes and phenotypically similar species, particularly within the last decade; and many may have already existed in the pet trade, and are imported under synonyms. Some examples include the multiple attempts to split the *Calotes mystaceus* complex within *Agamidae* [38,39], descriptions of new species in the *Hemidactylus* genus [40,41] and within the *Gekko* genus in *Gekkonidae* [42,43]. Therefore, new descriptions may be driving demand for lizards, or alternatively, may be inflating the number of new species being imported due to new reporting classifications based on species delimitation. This in turn will cause further fluctuation in reported species, particularly when subsequent studies suggest fewer species than earlier delimitations denote, thus reducing the oversplitting of species [44]. Alternatively, the observed trends here may suggest that there has been a push towards the perceived rare and unique within the pet trade in the US. This has been demonstrated within the European pet trade where advertisements and general trade patterns suggest a propensity for the trade of precepted “rare” species [22]. Species have been shown to enter the pet trade very shortly after the description of a new species (sometimes within a few months; [22,12]. Although I did not investigate species descriptions and subsequent time to importation, a similar trend may be happening in this circumstance.

The low number of imports during the later portion of the recording period could be due to a number of reasons. The first theory is that this is further evidence for a drive towards rare and unique species, where lower number of perceived rare species are warranted over quantity. Alternatively, this may be driven by captive-breeding pressures where breeding quantities are becoming sufficient to supply domestic demand especially as many of the most imported species are commonly kept, or historically kept, in captivity [45]. Hopefully there begins to be an established breeding population of rare species which reduces the need for new wild-sourced imports. Additionally, priorities for captive-breeding may have also changed to produce expensive phenotypic “morphs” such as seen in ball (royal) pythons, corn snakes, and bearded dragons [46,45,47]. This may hold particularly true for agamid lizards where I observed a shift from wild-caught individuals to captive-bred imports, and the imports were dominated by bearded dragons (though high numbers of other genera are evident). It can also be particularly difficult to breed rare, unique, and specialist species in captivity due to environmental conditions which are difficult to emulate in captive settings. For example, *Smaug mossambicus* appear to require seasonal temperature fluctuations to promote breeding activity [48]. Moreover, there is a paucity in important information needed for successful breeding such as in-situ habitat conditions, captive breeding success, and incubation conditions [49,50]. Additional domestic data could be critical for understanding how captive breeding interacts with the import demand demonstrated here. Furthermore, observed changed in import patterns may also be a consequence of updated reporting requirements from both exporting countries, and at ports of entry into the US, leading to under-reporting issues [51].

There is a dominance of wild-sourced individuals being imported across the study period (61.7%), particularly within some families specifically, such as *Gekkonidae* (96.2%). However, some family-level imports are dominated by captive sources (e.g., *Iguanidae*), or have demonstrated a trend towards captive-breeding instead of sourcing from wild populations (e.g., *Agamidae*). This latter pattern may demonstrate that an increase in captive-breeding efforts could potentially mitigate the reliance on wild populations to meet the demands of the pet trade. Although the general patterns of lizard imports suggests a lower demand for lizard imports into the US altogether, there may still be high demand for specific groups. The proportion of imported lizards that were comprised of agamids increased over time, revealing that increased demand over time. However, there has been an evident shift to captive-breeding over wild-caught individuals within this family. Captive-breeding could thus reduce the pressures of exploitation on lizard populations, largely reducing several ethical and conservation impacts that the pet trade may be having globally.

### 4.2 What is driving lizard imports?

The substantial importation of lizards into the US is likely driven by a combination of ecological, economic, and aesthetic factors, particularly those related to the characteristics of the most heavily imported families: *Gekkonidae*, *Agamidae*, and *Iguanidae*. These families generally consist of small- to medium-sized lizards – though large species of agamids and iguanids exist – which can make them manageable for captivity; potentially increasing desirability for the pet trade. However, a recent analysis on the trade susceptibility of geckos demonstrated that trade desirability is strongly associated with morphological traits, with larger species preferentially targeted [52]. Diverse morphology, unique ornamentations, and vibrant colours could further increase their appeal. For example, in *Agamidae*, *Chlamydosaurus kingii* possess frills used for intraspecific communication and predator deterrence [53], while *Hydrosaurus* species have unique and characteristically high dorsal crests [54]. Such features align with consumer preferences for visually distinctive and charismatic species (and often perceived rarity), potentially increasing the appeal to private collectors and vendors [55].

Families such as *Gekkonidae* and *Agamidae* are characterized by ecological adaptability and high species diversity, particularly owed to constant and novel species delimitation. This facilitates the availability of new and perceived-to-be-rare species. Thus, phylogenetically distinct species may be more susceptible to trade [34,52,56]. Vibrant colour patterns, such as the brilliant hues found in many agamid and gekkonid lizards, further contribute to their appeal, particularly as display species. The demand could also be shaped by their affordability relative to larger or more specialized reptiles; or conversely, their expense as a business investment if breeding populations can be established. Collectively, these attributes make the lizards from these families drive the US lizard import market, suggesting a mix of practical, aesthetic, and market-driven motivations. However, further research, particularly involving the socioeconomic drivers of the pet trade, could elucidate the main stimuli for lizard imports.

Importations investigated here were primarily of green iguanas, constituting 21.6% of all importations by quantity. This is consistent with previous investigations into reptile trade on a global scale, where a decrease over time was also observed [57]. Green iguanas have historically been farmed in Central America for meat due to their believed nutritional and medicinal properties [58]. A demand for iguana consumption may also explain the high imports observed here, particularly earlier within the LEMIS reporting. After the green iguana, imports are largely of bearded dragons (8.2%) and Indochinese water dragons (7.7%). Both species are commonly kept in captivity, and imports are likely driven by the demand for the pet trade, particularly when considering the “morph” market for bearded dragons [46,59,45,47].

### 4.3 Distributions and hotspots of imported lizards

**Fig 4:**
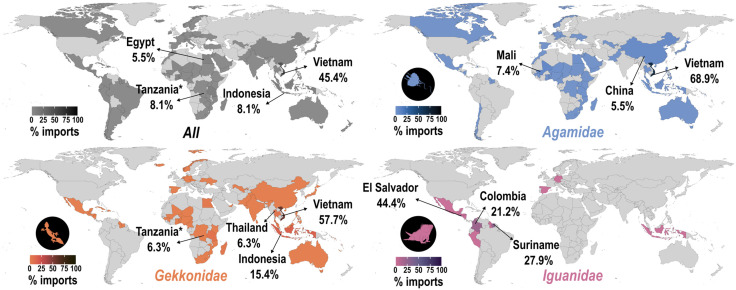
Origin maps demonstrating the percentage of wild-caught lizards imported from each country into the United States according to the United States Fish and Wildlife Service’s Law Enforcement Management Information System (LEMIS) dataset between 2000-2022. Dark grey represents imports for all lizard species, orange, blue, and purple depict lizards in the family *Gekkonidae*, *Agamidae*, and *Iguanidae*, respectively. Tanzania* = United Republic of Tanzania.

**Fig 5:**
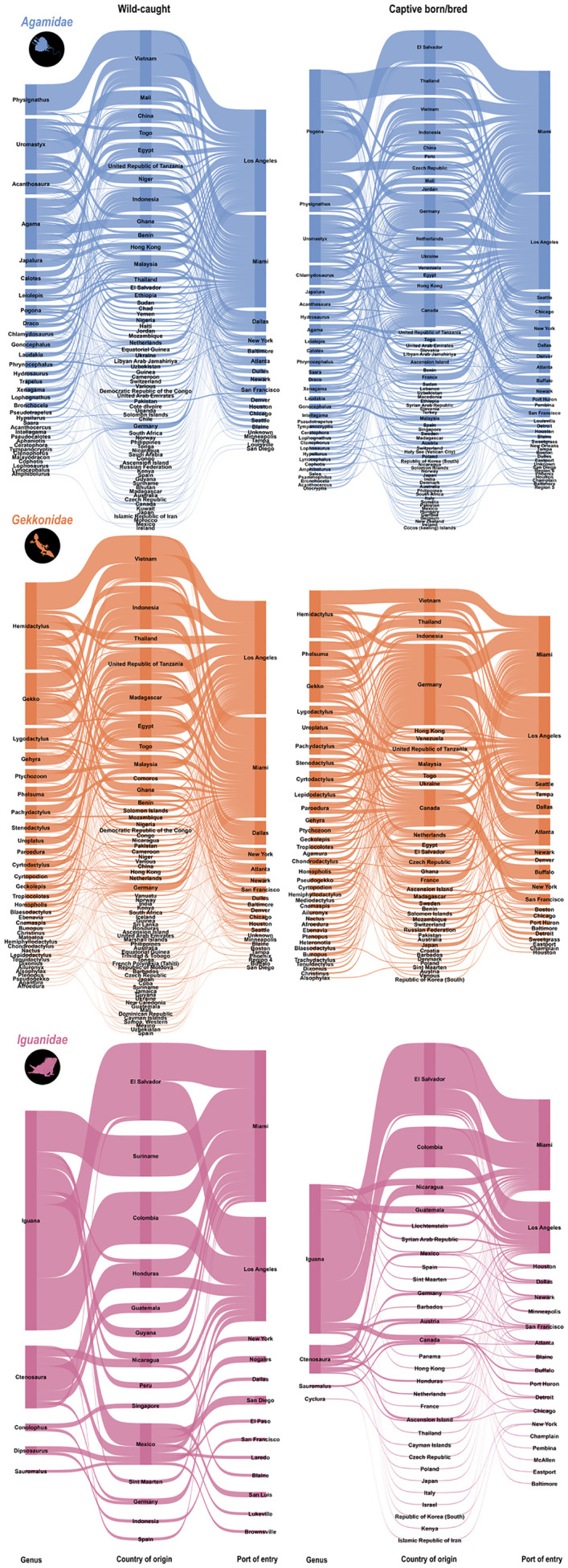
Sankey diagrams summarising import numbers, and routes into the United States according to the United States Fish and Wildlife Service’s Law Enforcement Management Information System (LEMIS) dataset between 2000–2022. Left nodes show the genera, leading into country of origin, and port of entry from left to right, respectively. The weight of the flow between nodes highlights the number of imports. Blue, orange, and pink diagrams show *Agamidae*, *Gekkonidae*, and *Iguanidae*, respectively. Top diagrams show imports from all wild-caught individuals, and bottom diagrams show all individuals sourced from captivity.

Almost half of the wild-sourced lizards were imported from Vietnam (45.4%). Far fewer of the imports came from other countries, with most other imports originating from Indonesia (8.1%), the United Republic of Tanzania (7.9%), Egypt (5.5%) and Togo (4.4%; Fig 4). These patterns appear to be largely driven by the import patterns across the two most imported lizard families where 68.9%, 2.5%, and 2.3% of agamid imports were from Vietnam, Togo, and Egypt, respectively, and 57.7%, 15.4%, and 6.3% of gekkonid imports were from Vietnam, Indonesia, and the United Republic of Tanzania, respectively. Imports originating in the United Republic of Tanzania are particularly interesting, specifically because they are shown to be the second highest export country of wild individuals into the US despite an export ban which came into effect in 2016 [60]. The data presented here for this country therefore contains imports between 2000–2016. The imports sourced from captivity tell a different story, where most imports originated in El Salvador (59.2%), followed by Colombia (16.8%), Nicaragua (8.1%), Thailand (5.3%), and Vietnam (2.8%). These summaries largely demonstrate that the number of export countries, and ports of entry, increased substantially when including import data where species do not have provenance (Fig 5). This is congruent with a recent assessment of the geographic origins of LEMIS imports, where Vietnam was shown to be the primary importer of reptiles (by quantity), followed by El Salvador [61]. In addition, Vietnam was shown to export very few captively-sourced individuals, unlike El Salvador that exports almost exclusively individuals sourced from captivity [61]. I observed the same patterns during this review of lizard imports into the US.

Future investigations into sustainable harvesting practices may be warranted in Southeast Asia, central and eastern Africa, and multiple countries in Central and South America (Fig 4). *Gekkonidae* and *Agamidae* both appear to have the highest collection pressures from Vietnam, which largely drives the trend across all lizard families (Fig 4). Vietnam has relatively high endemism, many of which are lizards, with one of the major threats to many of these species being exploitation [25]. Several African countries, particularly those on the mainland such as the United Republic of Tanzania, Togo, and Egypt, were primary trading hubs for live snake trade [28], and my study suggests that additional exploitation/trade pressures on reptile species may be acting within these African countries.

### 4.4 Threat of novel invasive species

**Fig 6:**
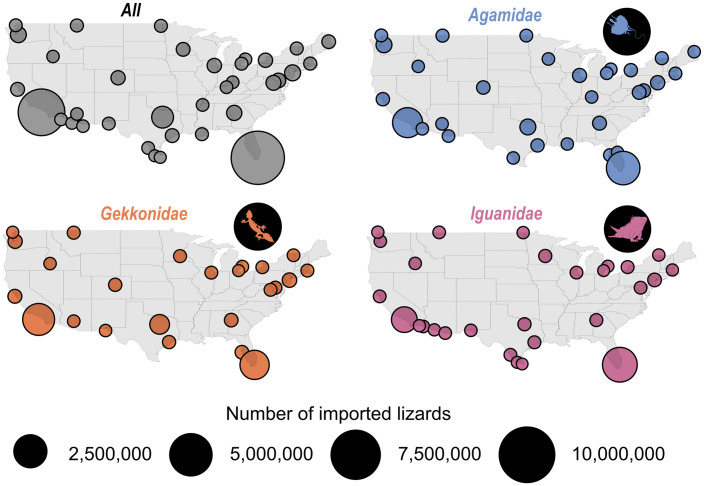
Number of imported lizard species to each port of entry recorded in the United States Fish and Wildlife Service’s Law Enforcement Management Information System (LEMIS) dataset between 2000-2022. Grey points represent numbers for all imported lizard species, orange, blue, and purple depict lizards in the family *Gekkonidae*, *Agamidae*, and *Iguanidae*, respectively. The size of the point represents the number of lizards imported to each port of entry.

Although some non-native reptile species have had detrimental effects on native ecosystems, they generally are not considered as some of the worst invasive species when summarizing across the tree of life, and global examples. In fact, only two reptiles are listed as one of the 100 worst invasive species by the IUCN [62], and neither of those species are lizards. There are some clear exceptions for reptile species, such as the Brown Tree Snake (*Boiga irregularis*) in Guam [63] and the Burmese Python in south Florida [64]. Miami, Florida is highlighted as the primary port of entry into the United States for lizard imports (Fig 6). South Florida is a hotspot for invasive reptiles, with several studies highlighting the establishment and pervasiveness of several non-native lizard species (e.g., [65,66,67]). Some of the non-native and invasive lizards in south Florida are species found within the three most imported families: *Gekkonidae* [68], *Agamidae* [69,70], and *Iguanidae* [71]. However, several species of non-native lizards found in south Florida are also from families that have had a relatively low number of imports (proportionally) throughout the study period; such as *Teiidae* [72] and *Anolidae* (S1 Fig; [73,74]). These latter examples highlight the high risk and propensity for lizard invasions even without high volumes of trade. With south Florida seemingly a major port of entry for lizard species (and likely other species), its subtropical climate, an ongoing and historically popular location for captive wildlife hobbyists, alongside an area regularly struck by hurricanes and other inclement weather that can instigate the unwelcome release of captive animals, there is no surprise that this area has been a haven for the establishment of non-native reptile species [75]. This invasion history may have been largely driven by the importation of lizards into Miami as a major port of entry, and could continue to be threatened by subsequent importations, particularly with the frequency of extreme weather events in the state. Engeman et al. [75] provide a review of prominent non-native reptiles to Florida and five of the six major species highlighted are lizard species.

The second most popular port of entry is Los Angeles, California (Fig 6). There are several known populations of non-native and invasive lizards to southern California [76,77]. Although the prevalence of invasive lizards and other reptiles is not at the same level as can be seen within south Florida ecosystems, the south California area could also be a potential hotspot for future introductions. I believe that this could be driven by the same factors acting on south Florida; namely, an apparent hub for lizard imports into the country, a warm climate, and a relatively high rate of extreme weather events. More locations in the US could be at risk of invasion by lizards and other reptiles, particularly under future climate change scenarios [78].

### 4.5 Limitations

The limitations of the LEMIS data have been discussed across several other studies [7,26,27,8]. Goodman and Kolby [26] specifically discuss the data gaps that can exist within LEMIS data, and this can be due to a number of reasons, including several exceptions to reporting requirements to USFWS. Weissgold [27] provides a more detailed overview of the shortcomings to LEMIS data highlighting that general inaccuracies, missing entries, and lack of standardization is commonplace; and additionally, recommends that these are discussed with subsequent use of LEMIS data. Since the lizard species recorded within the LEMIS dataset (investigated here) only represent less than 15% of the overall lizard diversity [19], this would suggest overall that the US pet trade is having a relatively small impact to lizard populations. However, I fear that the import data for lizards into the United States is substantially underrepresenting the true number of imports, particularly concerning genera and species diversity [12]. Particularly because the LEMIS data investigated here focuses only on imports into the US, and although this contains information about exports from other countries, the import demand from these countries is unavailable via LEMIS alone (and limited via other means).Thus, this study will likely be considerably underestimating trade at a global scale. Some of the limitations are simply due to identification errors at the ports of entry, where several species are only identified to the genus level, or attributed to a different species than actual. However, there are several species and genera that I anecdotally know to have been imported into the US from wild populations within the data collection timeframe. Examples of this includes the genus *Calotes (Agamidae*) where the only confirmed species being imported are *C. calotes*, *C. mystaceus*, *C. versicolor*, and *C. emma*, whereas there are several other species that have been imported to the United States, such as *C. bachae* and *C. pethiyagodai* (the former of which can often be imported under the name *C. versicolor* or *C. mystaceus*)*.* Also in *Agamidae*, there are no reported imports of *Hypsilurus magnus*, which is seemingly a relatively commonly imported species to the US within this genus. These evident omissions could be due to USFWS exceptions [26], but more likely represent cases of inaccurate identification of species.

[8] provide some suggestions for improving the accuracy, and thus applicability, of LEMIS data. These include the accurate identification of species using recent and widely accepted naming conventions, standardising recording units, automating processes for data reassurances and quality control, and making data open-access and publicly available in a timely manner. The correct identification of species can be challenging for non-experts, particularly with an ever-changing and updating taxonomic database of species names. The LEMIS recording system may benefit from multiple panels of taxa experts, potentially linked through an app or other communication system, that would allow timely identification of unique and unfamiliar species. For lizards specifically, the number of new species being imported into the US continued to rise between 2000–2022. This likely reflects the rapid rate of species delimitation within this diverse taxon, and thus the importance of having expert consultations to move towards an accurate database of trade. In principle, the process of creating such panels would be logistically and economically challenging. However, an identification system similar to that undertaken by iNaturalist could be adopted which could facilitate the technicality behind this. Zoos and academic facilities across the US are well-situated to collaborate with USFWS to offer guidance on species identification, or computer-aided identification systems. While there are recent moves towards targeting sustainable wildlife trade such as the debut of the Kunming-Montreal Global Biodiversity Framework in 2022 [56], and those more taxon-focused goals within the US specifically, such as the formation of the Collaborative to Combat Illegal Turtle Trade (CCITT; [79]), there remains a lack of policy, governance, and reporting measures on a global level that allow the extent of wildlife trade to be accurately evaluated. Several studies using LEMIS data have bolstered this data with information from the Convention on International Trade in Endangered Species (CITES) which in combination provides a more complete picture of trade in the US [12,8]. However, there is a wealth of data available in local markets and via online assessments which may be better suited to filling in the evident data gaps from reporting programs such as LEMIS and CITES, especially as many species (particularly reptiles) are exempt from reporting requirements. Further research should use all available data which in turn can help overcome the shortcomings of the LEMIS data, particularly studies that provide an online assessment of domestic trade [28,80].

During my review of each genus and associated “country_origin” data, I found several instances of inconsistent provenance that are commonly found across the LEMIS dataset [61]. Here, I highlight some notable examples. The genus *Conolophus*, which are endemic to the Galapagos Islands, many of which were listed as wild-caught yet originated from Indonesia and Singapore. This is congruent with a recent study highlighting that Galapagos iguanas have been previously laundered into international trade [81]. All of the *Heloderma* imported from Indonesia are also listed as wild-caught, even though this genus is distributed in North and Central America. Similarly, *Cnemidophorus* and *Tropidurus* are listed as wild-caught from Nigeria and Kenya, respectively, despite being endemic to the Americas. Some of the inconsistencies discovered may highlight trafficking routes by poachers, particularly those origins that are adjacent to known species distributions, or general inaccuracies (such as species misidentification), or could highlight false entries to LEMIS (to obscure true species imports). Additional work is needed to quantify the trends across the origin data, and what implications these mismatches have for lizards, and other wildlife. These entries may also contain data where third-party countries are listed as country of origin despite being used to temporarily hold individuals in transit [81,61]. These data may be purposefully obscured during import reporting, thus highlighting export routes that may be indicative of both regulated legal trade, and unregulated illegal trade [61]. My summary of the lizard import data does not allow me to evaluate the legality of much of the data. However, specific records, such as the records of *Conolophus* imported into the US in 2000 and 2010 from Indonesia and Singapore, respectively — although this should be impossible [81] — suggest that species or genus-specific analyses may uncover illegal importations into the US. Further work could further investigate species where export suspensions occur for their country of origin, such as some species in Madagascar, and across the United Republic of Tanzania.

The data cleaning effort by [8], and subsequently making LEMIS data open-access, will greatly improve the accessibility, usefulness, and applicability of trade data in the United States (and global impacts thereof). As highlighted in this study, there remains errors in this data that can still be amended to further improve the utility and accuracy of this data (although many of the issues are deeper-rooted than that; [27,26]. This further strengthens the importance of subsequent studies performing a “deep-dive” of the data by using taxonomic subsets to investigate patterns of trade. This will facilitate the identification of additional errors and taxonomic trends so that we can begin moving towards a more accurate holistic dataset for future use.

### 4.6 Overview, implications, and conclusions

The findings of this study underscore the large scale and diversity of lizard imports into the United States, emphasizing critical patterns and their implications for wildlife conservation, while likely underrepresenting the true magnitude of lizard trade. I found that there was an increase in the number of species, despite an overall decline in total individuals imported. These patterns likely reflect taxonomic revisions, changes in reporting and misidentification at ports of entry, and genuine shifts in trade owed to demand and animal sourcing (e.g., wild-caught vs captive-bred). From 2000 to 2022, there was a prevalence for wild-sourced lizard imports (61.7%) highlighting broader challenges in balancing commercial trade with sustainable practices. Of particular note is the dominance of imports from *Gekkonidae*, *Agamidae*, and *Iguanidae*, each presenting unique ecological and conservation concerns. For instance, while captive breeding appears to play a large role in *Iguanidae* and an increasing role in *Agamidae* (Fig 2), the sustained reliance on wild-caught individuals in other families, such as *Gekkonidae*, suggests vulnerability of numerous populations to overexploitation. The patterns also suggest that keepers are failing to establish captive populations of many prominent species (particularly in *Gekkonidae*; Fig 2) which in turn is indicative of an unsustainable industry. Future work could investigate the sustainability of harvesting native lizards from several of the hotspots highlighted in this study. However, there does appear to be a temporal shift from wild-sourced to captive-sourced individuals for agamid lizards, demonstrating that captive breeding could mitigate the impacts of exploitation due to the pet trade in specific groups.

This data review also highlights potential geographic biases in lizard importation, identifying key hotspots of collection and areas that may be under increased pressure from overharvesting. Areas in Southeast Asia, Central America, and Africa emerge as critical regions for targeted conservation interventions, where improved monitoring and regulation could mitigate the risks associated with underregulated trade and poaching activity. I suggest that additional work is undertaken to understand the impacts of trade to native species in Vietnam specifically, where most of the wild-caught lizard imports originate, and considerable exploitation pressures are acting across taxa [25]. In addition, previous evidence highlighting the propensity for rare and newly described species to enter the trade shortly after discovery, and the suggestive trends from this study that collection favours rare species over time, raises ethical and ecological questions about the long-term impact of such practices on biodiversity [12,22]. Future studies could benefit from an expanded focus on the intersection of species description timelines, market trends, and conservation outcomes for lizard species to bolster previous work [12,17].

My study also highlights notable data limitations within the LEMIS database, such as species misidentifications and underreporting, which likely obscures the true scale of trade impacts. Additionally, the LEMIS data does not capture the propensity of illegal trade into the US, which is likely difficult to quantify but a prevalent problem [82,83]. Addressing data gaps through enhanced data standardization and reporting requirements is essential to refine conservation strategies and future policy frameworks. Lastly, my findings also demonstrate arrival pathways that may need to be considered in future horizon-scanning methodology for some of the major ports of entry highlighted here [84]; namely, southern Florida and California [34,85]. Together, these efforts can pave the way for a more balanced coexistence between trade demands and biodiversity conservation.

## Supporting information

S1 Table Dead lizard records imported into the United States. Number of lizards imported into the United States that were recorded as dead according to the United States Fish and Wildlife Service’s Law Enforcement Management Information System (LEMIS) dataset between 2000 and 2022. These import data were removed from data summaries.
(DOCX)

S2 Table Removed lizard records originating from the United States. Records of imported lizards into the United States according to the United States Fish and Wildlife Service’s Law Enforcement Management Information System (LEMIS) dataset between 2000 and 2022 that recorded the country of origin as the United States. These import data were removed from data summaries.
(DOCX)

S3 Table Source information for imported lizards into the United States. Source of importation into the United States for all imported genera across represented families, excluding *Gekkonidae*, *Agamidae*, and *Iguanidae* (Table 1) according to the United States Fish and Wildlife Service’s Law Enforcement Management Information System (LEMIS) dataset between 2000 and 2022.
(DOCX)

S1 Fig Percentage of imported lizards represented at the family level. The percentage of imported lizards into the United States that represented each family between 2000 and 2022 according to the United States Fish and Wildlife Service’s Law Enforcement Management Information System (LEMIS) dataset. Each family is labelled above each plot.
(DOCX)
