# Supplementary material for: Saur and decline: Patterns in lizard imports to the US (2000–2022)
Source: PLoS One. 2025 Oct 22;20(10):e0333746. doi: 10.1371/journal.pone.0333746 (PMC12543155; doi:10.1371/journal.pone.0333746)
Supplement: S1 Table — Number of lizards imported into the United States that were recorded as dead according to the United States Fish and Wildlife Service’s Law Enforcement Management Information System (LEMIS) dataset between 2000 and 2022. These import data were removed from data summaries. (DOCX) [file pone.0333746.s001.docx]

Table S1. Dead lizard records imported into the United States. Number of lizards imported into the United States that were recorded as dead according to the United States Fish and Wildlife Service’s Law Enforcement Management Information System (LEMIS) dataset between 2000 and 2022. These import data were removed from data summaries.

| **Genus** | **Species** | **Imported lizards** |
| --- | --- | --- |
| *Agamidae* | | |
| *Agama* | Agama agama | 33 |
| *Calotes* | Calotes | 7 |
| *Pogona* | Pogona vitticeps | 89 |
| *Uromastyx* | Uromastyx geyri | 4 |
| *Uromastyx* | Uromastyx ocellata | 46 |
| *Amphisbaenidae* | | |
| *Amphisbaena* | *Amphisbaena* | 4 |
| *Anolidae* | | |
| *Anolis* | *Anolis* | 23 |
| *Chamaeleonidae* | | |
| *Bradypodion* | *Bradypodion spinosum* | 2 |
| *Bradypodion* | *Kinyongia fischeri* | 2 |
| *Brookesia* | *Brookesia therezieni* | 1 |
| *Calumma* | *Archaius tigris* | 1 |
| *Chamaeleo* | *Chamaeleo* | 29 |
| *Chamaeleo* | *Chamaeleo calyptratus* | 104 |
| *Chamaeleo* | *Chamaeleo dilepis* | 6 |
| *Chamaeleo* | *Chamaeleo fuelleborni* | 4 |
| *Chamaeleo* | *Chamaeleo gracilis* | 1 |
| *Chamaeleo* | *Chamaeleo senegalensis* | 5 |
| *Chamaeleo* | *Trioceros cristatus* | 6 |
| *Chamaeleo* | *Trioceros melleri* | 1 |
| *Chamaeleo* | *Trioceros montium* | 15 |
| *Furcifer* | *Furcifer lateralis* | 1 |
| *Furcifer* | *Furcifer polleni* | 4 |
| *Kinyongia* | *Kinyongia tenuis* | 2 |
| *Rhampholeon* | *Rhampholeon spectrum* | 35 |
| *Trioceros* | *Trioceros hoehnelii* | 3 |
| *Trioceros* | *Trioceros jacksonii* | 5 |
| *Cordylidae* | | |
| *Cordylus* | *Cordylus* | 1 |
| *Eublepharidae* | | |
| *Aeluroscalabotes* | *Aeluroscalabotes felinus* | 6 |
| *Hemitheconyx* | *Hemitheconyx caudicinctus* | 12 |
| *Holodactylus* | *Holodactylus* | 30 |
| *Gekkonidae* | | |
| *Cyrtodactylus* | *Cyrtodactylus* | 41 |
| *Cyrtodactylus* | *Cyrtodactylus consobrinus* | 11 |
| *Cyrtodactylus* | *Cyrtodactylus pulchellus* | 11 |
| *Hemidactylus* | *Hemidactylus* | 1 |
| *Lygodactylus* | *Lygodactylus conraui* | 20 |
| *Paroedura* | *Paroedura* | 6 |
| *Paroedura* | *Paroedura bastardi* | 15 |
| *Uroplatus* | *Uroplatus ebenaui* | 4 |
| *Uroplatus* | *Uroplatus phantasticus* | 2 |
| *Uroplatus* | *Uroplatus sikorae* | 9 |
| *Iguanidae* | | |
| *Iguana* | *Iguana iguana* | 1 |
| *Scincidae* | | |
| *Mabuya* | *Mabuya* | 145 |
| *Tiliqua* | *Tiliqua gigas* | 2 |
| *Sphaerodactylidae* | | |
| *Sphaerodactylus* | *Sphaerodactylus* | 17 |
| *Varanidae* | | |
| *Varanus* | *Varanus* | 2 |
| *Varanus* | *Varanus doreanus* | 1 |
| *Varanus* | *Varanus prasinus* | 1 |
