# Supplementary material for: Saur and decline: Patterns in lizard imports to the US (2000–2022)
Source: PLoS One. 2025 Oct 22;20(10):e0333746. doi: 10.1371/journal.pone.0333746 (PMC12543155; doi:10.1371/journal.pone.0333746)
Supplement: S2 Table — Records of imported lizards into the United States according to the United States Fish and Wildlife Service’s Law Enforcement Management Information System (LEMIS) dataset between 2000 and 2022 that recorded the country of origin as the United States. These import data were removed from data summaries. (DOCX) [file pone.0333746.s002.docx]

Table S2. Removed lizard records originating from the United States. Records of imported lizards into the United States according to the United States Fish and Wildlife Service’s Law Enforcement Management Information System (LEMIS) dataset between 2000 and 2022 that recorded the country of origin as the United States. These import data were removed from data summaries.

| **Species** | **Number of individuals** | **Percentage of total (%)** |
| --- | --- | --- |
| *Abronia* | 20 | 0.11 |
| *Acanthosaura* | 6 | 0.03 |
| *Acanthosaura armata* | 6 | 0.03 |
| *Acanthosaura capra* | 35 | 0.2 |
| *Aeluroscalabotes felinus* | 6 | 0.03 |
| *Afroedura* | 1 | 0.01 |
| *Afroedura africana* | 4 | 0.02 |
| *Afroedura loveridgei* | 6 | 0.03 |
| *Ameiva chaitzami* | 30 | 0.17 |
| *Amphibolurus* | 7 | 0.04 |
| *Anolis* | 3 | 0.02 |
| *Anolis bartschi* | 1 | 0.01 |
| *Anolis bimaculatus* | 4 | 0.02 |
| *Anolis carolinensis* | 5274 | 29.77 |
| *Anolis cristatellus* | 12 | 0.07 |
| *Anolis equestris* | 80 | 0.45 |
| *Anolis ferreus* | 6 | 0.03 |
| *Anolis grahami* | 16 | 0.09 |
| *Anolis leachii* | 7 | 0.04 |
| *Anolis limifrons* | 1 | 0.01 |
| *Anolis lividus* | 2 | 0.01 |
| *Anolis marmoratus* | 23 | 0.13 |
| *Anolis occulatus* | 4 | 0.02 |
| *Anolis roquet* | 2 | 0.01 |
| *Anolis sabanus* | 8 | 0.05 |
| *Anolis sagrei* | 1673 | 9.44 |
| *Anolis smallwoodi* | 3 | 0.02 |
| *Anolis valencienni* | 2 | 0.01 |
| *Basiliscus* | 50 | 0.28 |
| *Basiliscus plumifrons* | 27 | 0.15 |
| *Basiliscus vittatus* | 56 | 0.32 |
| *Callisaurus draconoides* | 61 | 0.34 |
| *Chamaeleo calyptratus* | 503 | 2.84 |
| *Chlamydosaurus kingii* | 3 | 0.02 |
| *Chondrodactylus angulifer* | 17 | 0.1 |
| *Chondrodactylus turneri* | 4 | 0.02 |
| *Cnemidophorus* | 6 | 0.03 |
| *Cnemidophorus gularis* | 5 | 0.03 |
| *Coleonyx* | 29 | 0.16 |
| *Coleonyx brevis* | 54 | 0.3 |
| *Coleonyx elegans* | 8 | 0.05 |
| *Coleonyx mitratus* | 2 | 0.01 |
| *Cophosaurus texanus* | 6 | 0.03 |
| *Cophoscincopus durus* | 12 | 0.07 |
| *Correlophus ciliatus* | 121 | 0.68 |
| *Correlophus sarasinorum* | 19 | 0.11 |
| *Corucia zebrata* | 60 | 0.34 |
| *Corytophanes cristatus* | 6 | 0.03 |
| *Crotaphytus collaris* | 651 | 3.67 |
| *Crotaphytus insularis* | 51 | 0.29 |
| *Ctenosaura pectinata* | 68 | 0.38 |
| *Cyclura nubila* | 1 | 0.01 |
| *Cyrtodactylus elok* | 20 | 0.11 |
| *Cyrtodactylus peguensis* | 4 | 0.02 |
| *Dasia olivacea* | 5 | 0.03 |
| *Diplodactylus* | 59 | 0.33 |
| *Dipsosaurus dorsalis* | 149 | 0.84 |
| *Dixonius siamensis* | 4 | 0.02 |
| *Egernia* | 5 | 0.03 |
| *Eublepharis* | 7 | 0.04 |
| *Eublepharis angramainyu* | 4 | 0.02 |
| *Eublepharis macularius* | 2347 | 13.25 |
| *Eublepharis turcmenicus* | 1 | 0.01 |
| *Eumeces algeriensis* | 1 | 0.01 |
| *Eumeces laticeps* | 10 | 0.06 |
| *Eurydactylodes agricolae* | 2 | 0.01 |
| *Eurydactylodes vieillardi* | 1 | 0.01 |
| *Furcifer pardalis* | 145 | 0.82 |
| *Gambelia wislizenii* | 113 | 0.64 |
| *Gekko* | 100 | 0.56 |
| *Gekko Laurenti,* | 200 | 1.13 |
| *Gekko gecko* | 2 | 0.01 |
| *Gerrhosaurus* | 1 | 0.01 |
| *Gerrhosaurus major* | 2 | 0.01 |
| *Gonatodes* | 1 | 0.01 |
| *Gonatodes albogularis* | 1 | 0.01 |
| *Gonatodes annularis* | 1 | 0.01 |
| *Gonatodes caudiscutatus* | 4 | 0.02 |
| *Gonatodes ceciliae* | 2 | 0.01 |
| *Gonatodes concinnatus* | 1 | 0.01 |
| *Gonatodes machelae* | 5 | 0.03 |
| *Gonatodes vittatus* | 4 | 0.02 |
| *Goniurosaurus kuroiwae* | 11 | 0.06 |
| *Goniurosaurus lichtenfelderi* | 4 | 0.02 |
| *Goniurosaurus orientalis* | 2 | 0.01 |
| *Heloderma horridum* | 27 | 0.15 |
| *Heloderma suspectum* | 8 | 0.05 |
| *Hemidactylus* | 4 | 0.02 |
| *Hemidactylus festivus* | 2 | 0.01 |
| *Hemidactylus frenatus* | 900 | 5.08 |
| *Hemidactylus inexpectatus* | 1 | 0.01 |
| *Hemidactylus lemurinus* | 2 | 0.01 |
| *Hemidactylus leschenaultii* | 2 | 0.01 |
| *Hemidactylus mabouia* | 3 | 0.02 |
| *Hemidactylus masirahensis* | 6 | 0.03 |
| *Hemidactylus turcicus* | 4 | 0.02 |
| *Hemitheconyx caudicinctus* | 403 | 2.27 |
| *Iguana* | 1 | 0.01 |
| *Iguana iguana* | 27 | 0.15 |
| *Intellagama lesueurii* | 34 | 0.19 |
| *Lacerta* | 19 | 0.11 |
| *Laemanctus longipes* | 6 | 0.03 |
| *Lamprolepis smaragdina* | 100 | 0.56 |
| *Leiocephalus carinatus* | 6 | 0.03 |
| *Leiolepis belliana* | 10 | 0.06 |
| *Mniarogekko chahoua* | 23 | 0.13 |
| *Nephrurus* | 23 | 0.13 |
| *Nephrurus asper* | 9 | 0.05 |
| *Nephrurus deleani* | 3 | 0.02 |
| *Nephrurus laevissimus* | 5 | 0.03 |
| *Nephrurus stellatus* | 5 | 0.03 |
| *Nephrurus vertebralis* | 5 | 0.03 |
| *Nephrurus wheeleri* | 3 | 0.02 |
| *Ophisaurus ventralis* | 8 | 0.05 |
| *Pachydactylus* | 17 | 0.1 |
| *Pachydactylus capensis* | 3 | 0.02 |
| *Pachydactylus fasciatus* | 4 | 0.02 |
| *Pachydactylus latirostris* | 2 | 0.01 |
| *Pachydactylus mariquensis* | 5 | 0.03 |
| *Pachydactylus scutatus* | 2 | 0.01 |
| *Pachydactylus weberi* | 5 | 0.03 |
| *Paroedura bastardi* | 12 | 0.07 |
| *Paroedura picta* | 16 | 0.09 |
| *Paroedura stumpffi* | 12 | 0.07 |
| *Phrynosoma modestum* | 6 | 0.03 |
| *Phrynosoma platyrhinos* | 175 | 0.99 |
| *Physignathus cocincinus* | 371 | 2.09 |
| *Pogona* | 49 | 0.28 |
| *Pogona barbata* | 10 | 0.06 |
| *Pogona henrylawsoni* | 1 | 0.01 |
| *Pogona minor* | 12 | 0.07 |
| *Pogona vitticeps* | 757 | 4.27 |
| *Polychrus marmoratus* | 4 | 0.02 |
| *Pristurus carteri* | 62 | 0.35 |
| *Pseudogekko smaragdinus* | 1 | 0.01 |
| *Ptenopus* | 6 | 0.03 |
| *Ptenopus kochi* | 4 | 0.02 |
| *Ptychozoon kuhli* | 4 | 0.02 |
| *Ptyodactylus guttatus* | 12 | 0.07 |
| *Rhacodactylus* | 2 | 0.01 |
| *Rhacodactylus auriculatus* | 139 | 0.78 |
| *Rhacodactylus chahoua* | 31 | 0.17 |
| *Rhacodactylus ciliatus* | 165 | 0.93 |
| *Rhacodactylus leachianus* | 154 | 0.87 |
| *Rhacodactylus trachyrhynchus* | 5 | 0.03 |
| *Salvator merianae* | 1 | 0.01 |
| *Sauromalus* | 1 | 0.01 |
| *Sauromalus obesus* | 20 | 0.11 |
| *Sceloporus* | 13 | 0.07 |
| *Sceloporus jarrovii* | 16 | 0.09 |
| *Sceloporus magister* | 3 | 0.02 |
| *Sceloporus malachiticus* | 51 | 0.29 |
| *Sceloporus occidentalis* | 44 | 0.25 |
| *Sceloporus olivaceus* | 2 | 0.01 |
| *Sceloporus poinsettii* | 49 | 0.28 |
| *Scincella lateralis* | 2 | 0.01 |
| *Sphaerodactylus* | 16 | 0.09 |
| *Sphaerodactylus alphus* | 10 | 0.06 |
| *Sphaerodactylus elegans* | 1 | 0.01 |
| *Sphaerodactylus fantasticus* | 3 | 0.02 |
| *Sphaerodactylus nigropunctatus* | 15 | 0.08 |
| *Sphaerodactylus poindexteri* | 2 | 0.01 |
| *Sphaerodactylus roosevelti* | 2 | 0.01 |
| *Sphaerodactylus ruibali* | 2 | 0.01 |
| *Sphaerodactylus scaber* | 1 | 0.01 |
| *Stenodactylus mauritanicus* | 8 | 0.05 |
| *Takydromus sexlineatus* | 900 | 5.08 |
| *Tenuidactylus caspius* | 4 | 0.02 |
| *Thecadactylus oskrobapreinorum* | 15 | 0.08 |
| *Thecadactylus rapicauda* | 2 | 0.01 |
| *Tiliqua* | 2 | 0.01 |
| *Tiliqua gigas* | 1 | 0.01 |
| *Tiliqua scincoides* | 7 | 0.04 |
| *Trioceros hoehnelii* | 110 | 0.62 |
| *Trioceros jacksonii* | 6 | 0.03 |
| *Trioceros rudis* | 2 | 0.01 |
| *Tropidurus torquatus* | 4 | 0.02 |
| *Tupinambis teguixin* | 2 | 0.01 |
| *Underwoodisaurus* | 3 | 0.02 |
| *Uromastyx aegyptia* | 3 | 0.02 |
| *Uroplatus* | 2 | 0.01 |
| *Uroplatus henkeli* | 6 | 0.03 |
| *Urosaurus ornatus* | 16 | 0.09 |
| *Uta stansburiana* | 117 | 0.66 |
| *Varanus dumerilii* | 1 | 0.01 |
| *Varanus exanthematicus* | 100 | 0.56 |
| *Varanus indicus* | 140 | 0.79 |
| *Varanus niloticus* | 1 | 0.01 |
| *Varanus salvator* | 1 | 0.01 |
| *Varanus spinulosus* | 12 | 0.07 |
| *Xenosaurus grandis* | 20 | 0.11 |
