# Supplementary material for: Saur and decline: Patterns in lizard imports to the US (2000–2022)
Source: PLoS One. 2025 Oct 22;20(10):e0333746. doi: 10.1371/journal.pone.0333746 (PMC12543155; doi:10.1371/journal.pone.0333746)
Supplement: S3 Table — Source of importation into the United States for all imported genera across represented families, excluding Gekkonidae, Agamidae, and Iguanidae (Table 1) according to the United States Fish and Wildlife Service’s Law Enforcement Management Information System (LEMIS) dataset between 2000 and 2022. (DOCX) [file pone.0333746.s003.docx]

Table S3. Source information for imported lizards into the United States. Source of importation into the United States for all imported genera across represented families, excluding *Gekkonidae*, *Agamidae*, and *Iguanidae* (Table 1) according to the United States Fish and Wildlife Service’s Law Enforcement Management Information System (LEMIS) dataset between 2000 and 2022.

| **Genus** | | **Source** | | **Imported lizards** | | **Percentage of genus (%)** | |
| --- | --- | --- | --- | --- | --- | --- | --- |
| Amphisbaenidae | | | | | | |  |
| *Amphisbaena* | Wild | | 319 | | 100 | |  |
| Anguidae | | | | | | |  |
| *Abronia* | Captive-bred | | 219 | | 74.7 | |  |
| *Abronia* | Captive-born | | 21 | | 7.2 | |  |
| *Abronia* | Unknown | | 1 | | 0.3 | |  |
| *Abronia* | Wild | | 52 | | 17.7 | |  |
| *Anguis* | Captive-bred | | 13 | | 100 | |  |
| *Elgaria* | Wild | | 2 | | 100 | |  |
| *Gerrhonotus* | Captive-bred | | 7 | | 0.8 | |  |
| *Gerrhonotus* | Unknown | | 81 | | 8.8 | |  |
| *Gerrhonotus* | Wild | | 834 | | 90.5 | |  |
| *Mesaspis* | Captive-bred | | 412 | | 99.3 | |  |
| *Mesaspis* | Wild | | 3 | | 0.7 | |  |
| *Ophisaurus* | Captive-bred | | 1037 | | 10.1 | |  |
| *Ophisaurus* | Wild | | 9221 | | 89.9 | |  |
| *Pseudopus* | Wild | | 880 | | 100 | |  |
| Anniellidae | | | | | | |  |
| *Anniella* | Wild | | 108 | | 100 | |  |
| Anolidae | | | | | | |  |
| *Anolis* | Captive-bred | | 3080 | | 3.8 | |  |
| *Anolis* | Captive-born | | 62 | | 0.1 | |  |
| *Anolis* | Unknown | | 1492 | | 1.9 | |  |
| *Anolis* | Wild | | 75271 | | 93.8 | |  |
| Bipedidae | | | | | | |  |
| *Bipes* | Captive-bred | | 11 | | 100 | |  |
| Carphodactylidae | | | | | | |  |
| *Carphodactylus* | Captive-bred | | 18 | | 94.7 | |  |
| *Carphodactylus* | Wild | | 1 | | 5.3 | |  |
| *Nephrurus* | Captive-bred | | 605 | | 80.1 | |  |
| *Nephrurus* | Wild | | 150 | | 19.9 | |  |
| *Phyllurus* | Captive-bred | | 62 | | 72.1 | |  |
| *Phyllurus* | Wild | | 24 | | 27.9 | |  |
| *Saltuarius* | Captive-bred | | 90 | | 83.3 | |  |
| *Saltuarius* | Wild | | 18 | | 16.7 | |  |
| *Underwoodisaurus* | Captive-bred | | 242 | | 83.2 | |  |
| *Underwoodisaurus* | Captive-born | | 1 | | 0.3 | |  |
| *Underwoodisaurus* | Wild | | 48 | | 16.5 | |  |
| Chamaeleonidae | | | | | | |  |
| *Archaius* | Captive-bred | | 8 | | 100 | |  |
| *Bradypodion* | Captive-bred | | 667 | | 1.6 | |  |
| *Bradypodion* | Captive-born | | 137 | | 0.3 | |  |
| *Bradypodion* | Unknown | | 338 | | 0.8 | |  |
| *Bradypodion* | Wild | | 40959 | | 97.3 | |  |
| *Brookesia* | Ranching | | 30 | | 0.2 | |  |
| *Brookesia* | Unknown | | 9 | | 0.1 | |  |
| *Brookesia* | Wild | | 12551 | | 99.7 | |  |
| *Calumma* | Captive-bred | | 298 | | 4.7 | |  |
| *Calumma* | Captive-born | | 35 | | 0.5 | |  |
| *Calumma* | Ranching | | 12 | | 0.2 | |  |
| *Calumma* | Wild | | 6026 | | 94.6 | |  |
| *Chamaeleo* | Captive-bred | | 106442 | | 21.9 | |  |
| *Chamaeleo* | Captive-born | | 2045 | | 0.4 | |  |
| *Chamaeleo* | Ranching | | 156821 | | 32.3 | |  |
| *Chamaeleo* | Unknown | | 151 | | 0 | |  |
| *Chamaeleo* | Wild | | 219498 | | 45.2 | |  |
| *Furcifer* | Captive-bred | | 11082 | | 15.8 | |  |
| *Furcifer* | Captive-born | | 463 | | 0.7 | |  |
| *Furcifer* | Ranching | | 1160 | | 1.7 | |  |
| *Furcifer* | Unknown | | 40 | | 0.1 | |  |
| *Furcifer* | Wild | | 57340 | | 81.8 | |  |
| *Kinyongia* | Captive-bred | | 4278 | | 36.5 | |  |
| *Kinyongia* | Captive-born | | 175 | | 1.5 | |  |
| *Kinyongia* | Wild | | 7279 | | 62 | |  |
| *Palleon* | Wild | | 53 | | 100 | |  |
| *Rhampholeon* | Captive-bred | | 3088 | | 2.4 | |  |
| *Rhampholeon* | Ranching | | 31 | | 0 | |  |
| *Rhampholeon* | Unknown | | 31 | | 0 | |  |
| *Rhampholeon* | Wild | | 126649 | | 97.6 | |  |
| *Rieppeleon* | Captive-bred | | 106 | | 0.2 | |  |
| *Rieppeleon* | Captive-born | | 1 | | 0 | |  |
| *Rieppeleon* | Wild | | 66034 | | 99.8 | |  |
| *Trioceros* | Captive-bred | | 35976 | | 80.7 | |  |
| *Trioceros* | Captive-born | | 890 | | 2 | |  |
| *Trioceros* | Wild | | 7733 | | 17.3 | |  |
| Cordylidae | | | | | | |  |
| *Cordylus* | Captive-bred | | 1130 | | 1.7 | |  |
| *Cordylus* | Captive-born | | 38 | | 0.1 | |  |
| *Cordylus* | Wild | | 66209 | | 98.3 | |  |
| *Ouroborus* | Captive-bred | | 13 | | 72.2 | |  |
| *Ouroborus* | Unknown | | 5 | | 27.8 | |  |
| *Platysaurus* | Captive-bred | | 846 | | 8.3 | |  |
| *Platysaurus* | Unknown | | 373 | | 3.7 | |  |
| *Platysaurus* | Wild | | 8928 | | 88 | |  |
| *Pseudocordylus* | Captive-bred | | 4 | | 8.5 | |  |
| *Pseudocordylus* | Captive-born | | 25 | | 53.2 | |  |
| *Pseudocordylus* | Wild | | 18 | | 38.3 | |  |
| *Smaug* | Captive-bred | | 139 | | 83.7 | |  |
| *Smaug* | Captive-born | | 17 | | 10.2 | |  |
| *Smaug* | Unknown | | 4 | | 2.4 | |  |
| *Smaug* | Wild | | 6 | | 3.6 | |  |
| Corytophanidae | | | | | | |  |
| *Basiliscus* | Captive-bred | | 125165 | | 83.4 | |  |
| *Basiliscus* | Captive-born | | 1 | | 0 | |  |
| *Basiliscus* | Ranching | | 300 | | 0.2 | |  |
| *Basiliscus* | Unknown | | 1022 | | 0.7 | |  |
| *Basiliscus* | Wild | | 23410 | | 15.6 | |  |
| *Corytophanes* | Captive-bred | | 3530 | | 17.8 | |  |
| *Corytophanes* | Captive-born | | 43 | | 0.2 | |  |
| *Corytophanes* | Unknown | | 1049 | | 5.3 | |  |
| *Corytophanes* | Wild | | 15210 | | 76.5 | |  |
| *Laemanctus* | Captive-bred | | 194 | | 2.1 | |  |
| *Laemanctus* | Unknown | | 230 | | 2.5 | |  |
| *Laemanctus* | Wild | | 8804 | | 95.4 | |  |
| Crotaphytidae | | | | | | |  |
| *Crotaphytus* | Captive-bred | | 50 | | 5.9 | |  |
| *Crotaphytus* | Unknown | | 1 | | 0.1 | |  |
| *Crotaphytus* | Wild | | 796 | | 94 | |  |
| *Gambelia* | Wild | | 4 | | 100 | |  |
| Diplodactylidae | | | | | | |  |
| *Bavayia* | Captive-bred | | 117 | | 83.6 | |  |
| *Bavayia* | Wild | | 23 | | 16.4 | |  |
| *Correlophus* | Captive-bred | | 2318 | | 82.7 | |  |
| *Correlophus* | Captive-born | | 3 | | 0.1 | |  |
| *Correlophus* | Wild | | 482 | | 17.2 | |  |
| *Diplodactylus* | Captive-bred | | 282 | | 44.8 | |  |
| *Diplodactylus* | Captive-born | | 1 | | 0.2 | |  |
| *Diplodactylus* | Wild | | 347 | | 55.1 | |  |
| *Eurydactylodes* | Captive-bred | | 669 | | 86.2 | |  |
| *Eurydactylodes* | Captive-born | | 8 | | 1 | |  |
| *Eurydactylodes* | Wild | | 99 | | 12.8 | |  |
| *Lucasium* | Captive-bred | | 63 | | 92.6 | |  |
| *Lucasium* | Wild | | 5 | | 7.4 | |  |
| *Mniarogekko* | Captive-bred | | 26 | | 76.5 | |  |
| *Mniarogekko* | Wild | | 8 | | 23.5 | |  |
| *Naultinus* | Captive-bred | | 20 | | 87 | |  |
| *Naultinus* | Captive-born | | 3 | | 13 | |  |
| *Oedura* | Captive-bred | | 265 | | 38.1 | |  |
| *Oedura* | Captive-born | | 4 | | 0.6 | |  |
| *Oedura* | Wild | | 426 | | 61.3 | |  |
| *Rhacodactylus* | Captive-bred | | 9001 | | 65.2 | |  |
| *Rhacodactylus* | Captive-born | | 12 | | 0.1 | |  |
| *Rhacodactylus* | Unknown | | 8 | | 0.1 | |  |
| *Rhacodactylus* | Wild | | 4779 | | 34.6 | |  |
| *Strophurus* | Captive-bred | | 820 | | 85.4 | |  |
| *Strophurus* | Wild | | 140 | | 14.6 | |  |
| Diploglossidae | | | | | | |  |
| *Celestus* | Wild | | 104 | | 100 | |  |
| *Diploglossus* | Captive-bred | | 122 | | 46.7 | |  |
| *Diploglossus* | Confiscated or seized | | 1 | | 0.4 | |  |
| *Diploglossus* | Unknown | | 4 | | 1.5 | |  |
| *Diploglossus* | Wild | | 130 | | 49.8 | |  |
| Eublepharidae | | | | | | |  |
| *Aeluroscalabotes* | Captive-bred | | 491 | | 10 | |  |
| *Aeluroscalabotes* | Wild | | 4399 | | 89.8 | |  |
| *Coleonyx* | Captive-bred | | 63004 | | 78.8 | |  |
| *Coleonyx* | Captive-born | | 83 | | 0.1 | |  |
| *Coleonyx* | Ranching | | 266 | | 0.3 | |  |
| *Coleonyx* | Unknown | | 1122 | | 1.4 | |  |
| *Coleonyx* | Wild | | 15408 | | 19.3 | |  |
| *Eublepharis* | Captive-bred | | 27389 | | 76.6 | |  |
| *Eublepharis* | Captive-born | | 19 | | 0.1 | |  |
| *Eublepharis* | Ranching | | 1 | | 0 | |  |
| *Eublepharis* | Unknown | | 1 | | 0 | |  |
| *Eublepharis* | Wild | | 8313 | | 23.3 | |  |
| *Goniurosaurus* | Captive-bred | | 8110 | | 39.4 | |  |
| *Goniurosaurus* | Captive-born | | 14 | | 0.1 | |  |
| *Goniurosaurus* | Ranching | | 96 | | 0.5 | |  |
| *Goniurosaurus* | Wild | | 12339 | | 60 | |  |
| *Hemitheconyx* | Captive-bred | | 7118 | | 2.6 | |  |
| *Hemitheconyx* | Ranching | | 25109 | | 9.3 | |  |
| *Hemitheconyx* | Unknown | | 1681 | | 0.6 | |  |
| *Hemitheconyx* | Wild | | 234865 | | 87.2 | |  |
| *Holodactylus* | Captive-bred | | 230 | | 1 | |  |
| *Holodactylus* | Unknown | | 200 | | 0.9 | |  |
| *Holodactylus* | Wild | | 22433 | | 98.1 | |  |
| Gerrhosauridae | | | | | | |  |
| *Gerrhosaurus* | Captive-bred | | 910 | | 0.9 | |  |
| *Gerrhosaurus* | Ranching | | 64 | | 0.1 | |  |
| *Gerrhosaurus* | Unknown | | 1303 | | 1.3 | |  |
| *Gerrhosaurus* | Wild | | 95301 | | 97.7 | |  |
| *Tetradactylus* | Captive-bred | | 100 | | 100 | |  |
| *Tracheloptychus* | Captive-bred | | 20 | | 0.1 | |  |
| *Tracheloptychus* | Unknown | | 6 | | 0 | |  |
| *Tracheloptychus* | Wild | | 13543 | | 99.8 | |  |
| *Zonosaurus* | Captive-bred | | 50 | | 0.9 | |  |
| *Zonosaurus* | Ranching | | 60 | | 1 | |  |
| *Zonosaurus* | Unknown | | 36 | | 0.6 | |  |
| *Zonosaurus* | Wild | | 5701 | | 97.5 | |  |
| Gymnopthalmidae | | | | | | |  |
| *Arthrosaura* | Captive-bred | | 3 | | 100 | |  |
| *Bachia* | Captive-bred | | 17 | | 56.7 | |  |
| *Bachia* | Wild | | 13 | | 43.3 | |  |
| *Gymnophthalmus* | Captive-bred | | 13 | | 100 | |  |
| *Neusticurus* | Captive-bred | | 4 | | 30.8 | |  |
| *Neusticurus* | Wild | | 9 | | 69.2 | |  |
| *Potamites* | Captive-bred | | 6 | | 50 | |  |
| *Potamites* | Wild | | 6 | | 50 | |  |
| *Vanzosaura* | Wild | | 50 | | 100 | |  |
| Helodermatidae | | | | | | |  |
| *Heloderma* | Captive-bred | | 161 | | 49.4 | |  |
| *Heloderma* | Wild | | 165 | | 50.6 | |  |
| Hoplocercidae | | | | | | |  |
| *Enyalioides* | Captive-bred | | 3 | | 1.6 | |  |
| *Enyalioides* | Wild | | 179 | | 98.4 | |  |
| Lacertidae | | | | | | |  |
| *Acanthodactylus* | Captive-bred | | 216 | | 0.9 | |  |
| *Acanthodactylus* | Unknown | | 300 | | 1.2 | |  |
| *Acanthodactylus* | Wild | | 24678 | | 98 | |  |
| *Adolfus* | Captive-bred | | 107 | | 7.6 | |  |
| *Adolfus* | Wild | | 1300 | | 92.4 | |  |
| *Darevskia* | Captive-bred | | 112 | | 88.9 | |  |
| *Darevskia* | Wild | | 14 | | 11.1 | |  |
| *Eremias* | Captive-bred | | 1179 | | 1.8 | |  |
| *Eremias* | Ranching | | 500 | | 0.8 | |  |
| *Eremias* | Wild | | 64102 | | 97.4 | |  |
| *Gallotia* | Captive-bred | | 64 | | 90.1 | |  |
| *Gallotia* | Wild | | 7 | | 9.9 | |  |
| *Gastropholis* | Captive-bred | | 187 | | 93.5 | |  |
| *Gastropholis* | Wild | | 13 | | 6.5 | |  |
| *Heliobolus* | Wild | | 150 | | 100 | |  |
| *Holaspis* | Captive-bred | | 95 | | 1 | |  |
| *Holaspis* | Wild | | 9362 | | 99 | |  |
| *Lacerta* | Captive-bred | | 2453 | | 4.2 | |  |
| *Lacerta* | Ranching | | 500 | | 0.9 | |  |
| *Lacerta* | Unknown | | 1045 | | 1.8 | |  |
| *Lacerta* | Wild | | 54594 | | 93.2 | |  |
| *Latastia* | Captive-bred | | 255 | | 1 | |  |
| *Latastia* | Unknown | | 100 | | 0.4 | |  |
| *Latastia* | Wild | | 24507 | | 98.6 | |  |
| *Mesalina* | Wild | | 200 | | 100 | |  |
| *Nucras* | Captive-bred | | 2 | | 1.7 | |  |
| *Nucras* | Wild | | 114 | | 98.3 | |  |
| *Omanosaura* | Captive-bred | | 62 | | 83.8 | |  |
| *Omanosaura* | Wild | | 12 | | 16.2 | |  |
| *Pedioplanis* | Captive-bred | | 16 | | 100 | |  |
| *Podarcis* | Captive-bred | | 197 | | 80.1 | |  |
| *Podarcis* | Wild | | 49 | | 19.9 | |  |
| *Psammodromus* | Captive-bred | | 4 | | 100 | |  |
| *Takydromus* | Captive-bred | | 54159 | | 3.8 | |  |
| *Takydromus* | Captive-born | | 11 | | 0 | |  |
| *Takydromus* | Ranching | | 1000 | | 0.1 | |  |
| *Takydromus* | Unknown | | 700 | | 0 | |  |
| *Takydromus* | Wild | | 1359097 | | 96.1 | |  |
| *Timon* | Captive-bred | | 1294 | | 78 | |  |
| *Timon* | Wild | | 366 | | 22 | |  |
| *Zootoca* | Wild | | 20 | | 100 | |  |
| Lanthanotidae | | | | | | |  |
| *Lanthanotus* | Captive-bred | | 82 | | 85.4 | |  |
| *Lanthanotus* | Wild | | 14 | | 14.6 | |  |
| Leiocephalidae | | | | | | |  |
| *Leiocephalus* | Captive-bred | | 3 | | 0 | |  |
| *Leiocephalus* | Captive-born | | 6 | | 0 | |  |
| *Leiocephalus* | Unknown | | 16391 | | 7.7 | |  |
| *Leiocephalus* | Wild | | 197031 | | 92.3 | |  |
| Leiosauridae | | | | | | |  |
| *Enyalius* | Captive-bred | | 7 | | 100 | |  |
| *Pristidactylus* | Captive-bred | | 2 | | 100 | |  |
| *Liolaemus* | Captive-bred | | 15 | | 0.1 | |  |
| *Liolaemus* | Unknown | | 2100 | | 11.3 | |  |
| *Liolaemus* | Wild | | 16417 | | 88.6 | |  |
| *Phymaturus* | Captive-bred | | 12 | | 75 | |  |
| *Phymaturus* | Wild | | 4 | | 25 | |  |
| Opluridae | | | | | | |  |
| *Chalarodon* | Wild | | 8419 | | 100 | |  |
| *Oplurus* | Captive-bred | | 11 | | 0.1 | |  |
| *Oplurus* | Ranching | | 20 | | 0.2 | |  |
| *Oplurus* | Unknown | | 105 | | 1 | |  |
| *Oplurus* | Wild | | 10590 | | 98.7 | |  |
| Phrynosomatidae | | | | | | |  |
| *Petrosaurus* | Captive-bred | | 400 | | 73.4 | |  |
| *Petrosaurus* | Wild | | 145 | | 26.6 | |  |
| *Phrynosoma* | Captive-bred | | 117 | | 78 | |  |
| *Phrynosoma* | Unknown | | 3 | | 2 | |  |
| *Phrynosoma* | Wild | | 30 | | 20 | |  |
| *Sceloporus* | Captive-bred | | 221521 | | 68.1 | |  |
| *Sceloporus* | Ranching | | 1000 | | 0.3 | |  |
| *Sceloporus* | Unknown | | 5129 | | 1.6 | |  |
| *Sceloporus* | Wild | | 96835 | | 29.8 | |  |
| *Uma* | Wild | | 15 | | 100 | |  |
| *Urosaurus* | Wild | | 32 | | 100 | |  |
| *Uta* | Wild | | 3 | | 100 | |  |
| Phyllodactylidae | | | | | | |  |
| *Asaccus* | Captive-bred | | 8 | | 53.3 | |  |
| *Asaccus* | Wild | | 7 | | 46.7 | |  |
| *Gymnodactylus* | Wild | | 1951 | | 100 | |  |
| *Homonota* | Captive-bred | | 89 | | 14.7 | |  |
| *Homonota* | Captive-born | | 2 | | 0.3 | |  |
| *Homonota* | Ranching | | 37 | | 6.1 | |  |
| *Homonota* | Wild | | 476 | | 78.5 | |  |
| *Phyllodactylus* | Captive-bred | | 211 | | 6 | |  |
| *Phyllodactylus* | Wild | | 3298 | | 94 | |  |
| *Ptyodactylus* | Captive-bred | | 473 | | 0.9 | |  |
| *Ptyodactylus* | Ranching | | 1220 | | 2.4 | |  |
| *Ptyodactylus* | Unknown | | 200 | | 0.4 | |  |
| *Ptyodactylus* | Wild | | 49618 | | 96.3 | |  |
| *Tarentola* | Captive-bred | | 1667 | | 1.1 | |  |
| *Tarentola* | Captive-born | | 19 | | 0 | |  |
| *Tarentola* | Ranching | | 850 | | 0.5 | |  |
| *Tarentola* | Unknown | | 3720 | | 2.3 | |  |
| *Tarentola* | Wild | | 152099 | | 96 | |  |
| *Thecadactylus* | Captive-bred | | 169 | | 5.3 | |  |
| *Thecadactylus* | Unknown | | 269 | | 8.4 | |  |
| *Thecadactylus* | Wild | | 2767 | | 86.3 | |  |
| Polychrotidae | | | | | | |  |
| *Polychrus* | Captive-bred | | 55 | | 1 | |  |
| *Polychrus* | Ranching | | 20 | | 0.3 | |  |
| *Polychrus* | Unknown | | 311 | | 5.4 | |  |
| *Polychrus* | Wild | | 5324 | | 92.8 | |  |
| Pygopodidae | | | | | | |  |
| *Lialis* | Captive-bred | | 17 | | 0.8 | |  |
| *Lialis* | Captive-born | | 6 | | 0.3 | |  |
| *Lialis* | Wild | | 2081 | | 98.9 | |  |
| Scincidae | | | | | | |  |
| *Acontias* | Captive-bred | | 2 | | 0.1 | |  |
| *Acontias* | Wild | | 3598 | | 99.9 | |  |
| *Amphiglossus* | Captive-bred | | 2 | | 0.2 | |  |
| *Amphiglossus* | Wild | | 1086 | | 99.8 | |  |
| *Bellatorias* | Wild | | 123 | | 100 | |  |
| *Brachyseps* | Wild | | 51 | | 100 | |  |
| *Chalcides* | Captive-bred | | 717 | | 0.8 | |  |
| *Chalcides* | Unknown | | 1660 | | 1.8 | |  |
| *Chalcides* | Wild | | 87476 | | 97.4 | |  |
| *Cophoscincopus* | Captive-bred | | 200 | | 9 | |  |
| *Cophoscincopus* | Wild | | 2023 | | 91 | |  |
| *Corucia* | Captive-bred | | 2983 | | 37 | |  |
| *Corucia* | Captive-born | | 17 | | 0.2 | |  |
| *Corucia* | Unknown | | 310 | | 3.8 | |  |
| *Corucia* | Wild | | 4745 | | 58.9 | |  |
| *Cryptoblepharus* | Wild | | 975 | | 100 | |  |
| *Ctenotus* | Wild | | 50 | | 100 | |  |
| *Cyclodomorphus* | Captive-bred | | 1033 | | 88.8 | |  |
| *Cyclodomorphus* | Wild | | 130 | | 11.2 | |  |
| *Dasia* | Captive-bred | | 14 | | 0.2 | |  |
| *Dasia* | Wild | | 5488 | | 98 | |  |
| *Egernia* | Captive-bred | | 541 | | 42.4 | |  |
| *Egernia* | Captive-born | | 8 | | 0.6 | |  |
| *Egernia* | Wild | | 727 | | 57 | |  |
| *Emoia* | Captive-bred | | 533 | | 6.5 | |  |
| *Emoia* | Wild | | 7706 | | 93.5 | |  |
| *Eugongylus* | Captive-bred | | 79 | | 5.9 | |  |
| *Eugongylus* | Wild | | 1258 | | 94.1 | |  |
| *Eumeces* | Captive-bred | | 468 | | 0.6 | |  |
| *Eumeces* | Captive-born | | 30 | | 0 | |  |
| *Eumeces* | Ranching | | 300 | | 0.4 | |  |
| *Eumeces* | Unknown | | 860 | | 1.2 | |  |
| *Eumeces* | Wild | | 71634 | | 97.7 | |  |
| *Eutropis* | Captive-bred | | 67 | | 6.1 | |  |
| *Eutropis* | Wild | | 1039 | | 93.9 | |  |
| *Flexiseps* | Wild | | 25 | | 100 | |  |
| *Hemiergis* | Captive-bred | | 10 | | 100 | |  |
| *Isopachys* | Captive-bred | | 12 | | 70.6 | |  |
| *Isopachys* | Wild | | 5 | | 29.4 | |  |
| *Lamprolepis* | Captive-bred | | 1319 | | 10.8 | |  |
| *Lamprolepis* | Wild | | 10880 | | 89.2 | |  |
| *Lygosoma* | Captive-bred | | 6 | | 0.2 | |  |
| *Lygosoma* | Unknown | | 100 | | 4 | |  |
| *Lygosoma* | Wild | | 2368 | | 95.7 | |  |
| *Mabuya* | Captive-bred | | 2540 | | 1.1 | |  |
| *Mabuya* | Ranching | | 6953 | | 3.1 | |  |
| *Mabuya* | Unknown | | 3563 | | 1.6 | |  |
| *Mabuya* | Wild | | 207938 | | 94.1 | |  |
| *Madascincus* | Captive-bred | | 4 | | 13.8 | |  |
| *Madascincus* | Wild | | 25 | | 86.2 | |  |
| *Marisora* | Captive-bred | | 10 | | 100 | |  |
| *Mochlus* | Captive-bred | | 2050 | | 1.9 | |  |
| *Mochlus* | Ranching | | 8044 | | 7.5 | |  |
| *Mochlus* | Unknown | | 554 | | 0.5 | |  |
| *Mochlus* | Wild | | 96491 | | 89.9 | |  |
| *Morethia* | Wild | | 25 | | 100 | |  |
| *Panaspis* | Wild | | 132 | | 100 | |  |
| *Plestiodon* | Captive-bred | | 6 | | 100 | |  |
| *Prasinohaema* | Wild | | 100 | | 100 | |  |
| *Pygomeles* | Wild | | 402 | | 100 | |  |
| *Riopa* | Captive-bred | | 8 | | 0.8 | |  |
| *Riopa* | Wild | | 1000 | | 99.2 | |  |
| *Scincella* | Wild | | 235 | | 100 | |  |
| *Scincopus* | Captive-bred | | 259 | | 2.6 | |  |
| *Scincopus* | Ranching | | 396 | | 3.9 | |  |
| *Scincopus* | Wild | | 9403 | | 93.5 | |  |
| *Scincus* | Captive-bred | | 591 | | 0.9 | |  |
| *Scincus* | Ranching | | 351 | | 0.5 | |  |
| *Scincus* | Unknown | | 920 | | 1.3 | |  |
| *Scincus* | Wild | | 66790 | | 97.3 | |  |
| *Sphenomorphus* | Captive-bred | | 304 | | 51.1 | |  |
| *Sphenomorphus* | Wild | | 291 | | 48.9 | |  |
| *Tiliqua* | Captive-bred | | 11178 | | 20.2 | |  |
| *Tiliqua* | Captive-born | | 5049 | | 9.1 | |  |
| *Tiliqua* | Unknown | | 5 | | 0 | |  |
| *Tiliqua* | Wild | | 39107 | | 70.7 | |  |
| *Trachylepis* | Captive-bred | | 2133 | | 1.2 | |  |
| *Trachylepis* | Ranching | | 9150 | | 5.2 | |  |
| *Trachylepis* | Unknown | | 575 | | 0.3 | |  |
| *Trachylepis* | Wild | | 162874 | | 92.5 | |  |
| *Tribolonotus* | Captive-bred | | 1941 | | 5.9 | |  |
| *Tribolonotus* | Captive-born | | 484 | | 1.5 | |  |
| *Tribolonotus* | Wild | | 30364 | | 92.6 | |  |
| *Tropidophorus* | Captive-bred | | 95 | | 2 | |  |
| *Tropidophorus* | Captive-born | | 9 | | 0.2 | |  |
| *Tropidophorus* | Wild | | 4670 | | 97.8 | |  |
| *Tropidoscincus* | Wild | | 8 | | 100 | |  |
| Shinisauridae | | | | | | |  |
| *Shinisaurus* | Captive-bred | | 369 | | 96.3 | |  |
| *Shinisaurus* | Captive-born | | 5 | | 1.3 | |  |
| *Shinisaurus* | Wild | | 9 | | 2.3 | |  |
| Sphaerodactylidae | | | | | | |  |
| *Aristelliger* | Captive-bred | | 2 | | 100 | |  |
| *Gonatodes* | Captive-bred | | 20261 | | 92.4 | |  |
| *Gonatodes* | Wild | | 1659 | | 7.6 | |  |
| *Lepidoblepharis* | Captive-bred | | 2 | | 50 | |  |
| *Lepidoblepharis* | Wild | | 2 | | 50 | |  |
| *Pristurus* | Captive-bred | | 343 | | 85.8 | |  |
| *Pristurus* | Captive-born | | 4 | | 1 | |  |
| *Pristurus* | Wild | | 53 | | 13.2 | |  |
| *Quedenfeldtia* | Captive-bred | | 24 | | 92.3 | |  |
| *Quedenfeldtia* | Wild | | 2 | | 7.7 | |  |
| *Saurodactylus* | Captive-bred | | 58 | | 59.8 | |  |
| *Saurodactylus* | Wild | | 39 | | 40.2 | |  |
| *Sphaerodactylus* | Captive-bred | | 688 | | 40.9 | |  |
| *Sphaerodactylus* | Captive-born | | 1 | | 0.1 | |  |
| *Sphaerodactylus* | Unknown | | 2 | | 0.1 | |  |
| *Sphaerodactylus* | Wild | | 991 | | 58.9 | |  |
| *Teratoscincus* | Captive-bred | | 2937 | | 9.2 | |  |
| *Teratoscincus* | Unknown | | 100 | | 0.3 | |  |
| *Teratoscincus* | Wild | | 28907 | | 90.5 | |  |
| Teiidae | | | | | | |  |
| *Ameiva* | Captive-bred | | 25889 | | 21.9 | |  |
| *Ameiva* | Unknown | | 1730 | | 1.5 | |  |
| *Ameiva* | Wild | | 89995 | | 76.3 | |  |
| *Aspidoscelis* | Captive-bred | | 20496 | | 84 | |  |
| *Aspidoscelis* | Wild | | 3891 | | 16 | |  |
| *Callopistes* | Captive-bred | | 125 | | 2.1 | |  |
| *Callopistes* | Unknown | | 558 | | 9.6 | |  |
| *Callopistes* | Wild | | 5157 | | 88.3 | |  |
| *Cnemidophorus* | Captive-bred | | 3780 | | 8.5 | |  |
| *Cnemidophorus* | Unknown | | 1164 | | 2.6 | |  |
| *Cnemidophorus* | Wild | | 39629 | | 88.8 | |  |
| *Crocodilurus* | Captive-born | | 2 | | 100 | |  |
| *Dicrodon* | Wild | | 72 | | 100 | |  |
| *Dracaena* | Captive-bred | | 245 | | 5.9 | |  |
| *Dracaena* | Captive-born | | 3846 | | 92.9 | |  |
| *Dracaena* | Wild | | 48 | | 1.2 | |  |
| *Kentropyx* | Captive-bred | | 9 | | 56.2 | |  |
| *Kentropyx* | Wild | | 7 | | 43.8 | |  |
| *Pholidoscelis* | Wild | | 18 | | 100 | |  |
| *Salvator* | Captive-bred | | 5022 | | 100 | |  |
| *Teius* | Unknown | | 181 | | 22.5 | |  |
| *Teius* | Wild | | 623 | | 77.5 | |  |
| *Tupinambis* | Captive-bred | | 44889 | | 58.8 | |  |
| *Tupinambis* | Captive-born | | 2 | | 0 | |  |
| *Tupinambis* | Ranching | | 2588 | | 3.4 | |  |
| *Tupinambis* | Unknown | | 1 | | 0 | |  |
| *Tupinambis* | Wild | | 28856 | | 37.8 | |  |
| Trogonophidae | | | | | | |  |
| *Trogonophis* | Captive-bred | | 2 | | 20 | |  |
| *Trogonophis* | Captive-born | | 4 | | 40 | |  |
| *Trogonophis* | Wild | | 4 | | 40 | |  |
| Tropiduridae | | | | | | |  |
| *Microlophus* | Captive-bred | | 19 | | 1.5 | |  |
| *Microlophus* | Unknown | | 272 | | 21.1 | |  |
| *Microlophus* | Wild | | 997 | | 77.4 | |  |
| *Plica* | Unknown | | 208 | | 2.3 | |  |
| *Plica* | Wild | | 8917 | | 97.7 | |  |
| *Stenocercus* | Captive-bred | | 19 | | 2.2 | |  |
| *Stenocercus* | Unknown | | 136 | | 15.4 | |  |
| *Stenocercus* | Wild | | 728 | | 82.4 | |  |
| *Tropidurus* | Captive-bred | | 109 | | 0.1 | |  |
| *Tropidurus* | Ranching | | 76 | | 0.1 | |  |
| *Tropidurus* | Unknown | | 738 | | 1 | |  |
| *Tropidurus* | Wild | | 76296 | | 98.5 | |  |
| *Uracentron* | Confiscated or seized | | 2 | | 25 | |  |
| *Uracentron* | Wild | | 6 | | 75 | |  |
| *Uranoscodon* | Unknown | | 90 | | 1.6 | |  |
| *Uranoscodon* | Wild | | 5366 | | 98.4 | |  |
| Varanidae | | | | | | |  |
| *Varanus* | Captive-bred | | 17975 | | 2.3 | |  |
| *Varanus* | Captive-born | | 9854 | | 1.3 | |  |
| *Varanus* | Ranching | | 362260 | | 47.3 | |  |
| *Varanus* | Unknown | | 834 | | 0.1 | |  |
| *Varanus* | Wild | | 375293 | | 49 | |  |
| Xantusiidae | | | | | | |  |
| *Lepidophyma* | Captive-bred | | 543 | | 7.9 | |  |
| *Lepidophyma* | Unknown | | 750 | | 10.9 | |  |
| *Lepidophyma* | Wild | | 5586 | | 81.2 | |  |
| *Xantusia* | Captive-bred | | 3 | | 60 | |  |
| *Xantusia* | Wild | | 2 | | 40 | |  |
| Xenosauridae | | | | | | |  |
| *Xenosaurus* | Captive-bred | | 74 | | 43.3 | |  |
| *Xenosaurus* | Wild | | 97 | | 56.7 | |  |

*Captive-bred*: the successful breeding of lizards in captivity resulting in the birth/hatching of imported lizards; *captive-born*: imported individuals were born in captivity due to the importation of a gravid female lizard; wild: individuals were harvested from wild populations; *ranching*: imported lizards were harvested from the wild as eggs or juveniles and subsequently raised in captivity; *unknown*: source is unknown; c*ommerically bred*: individuals were produced in commercial settings; *confiscated or seized*: individuals were confiscated from poaching attempts or other illegal activities.
