# Supplementary material for: Saur and decline: Patterns in lizard imports to the US (2000–2022)
Source: PLoS One. 2025 Oct 22;20(10):e0333746. doi: 10.1371/journal.pone.0333746 (PMC12543155; doi:10.1371/journal.pone.0333746)
Supplement: S1 Fig — The percentage of imported lizards into the United States that represented each family between 2000 and 2022 according to the United States Fish and Wildlife Service’s Law Enforcement Management Information System (LEMIS) dataset. Each family is labelled above each plot. (DOCX) [file pone.0333746.s004.docx]

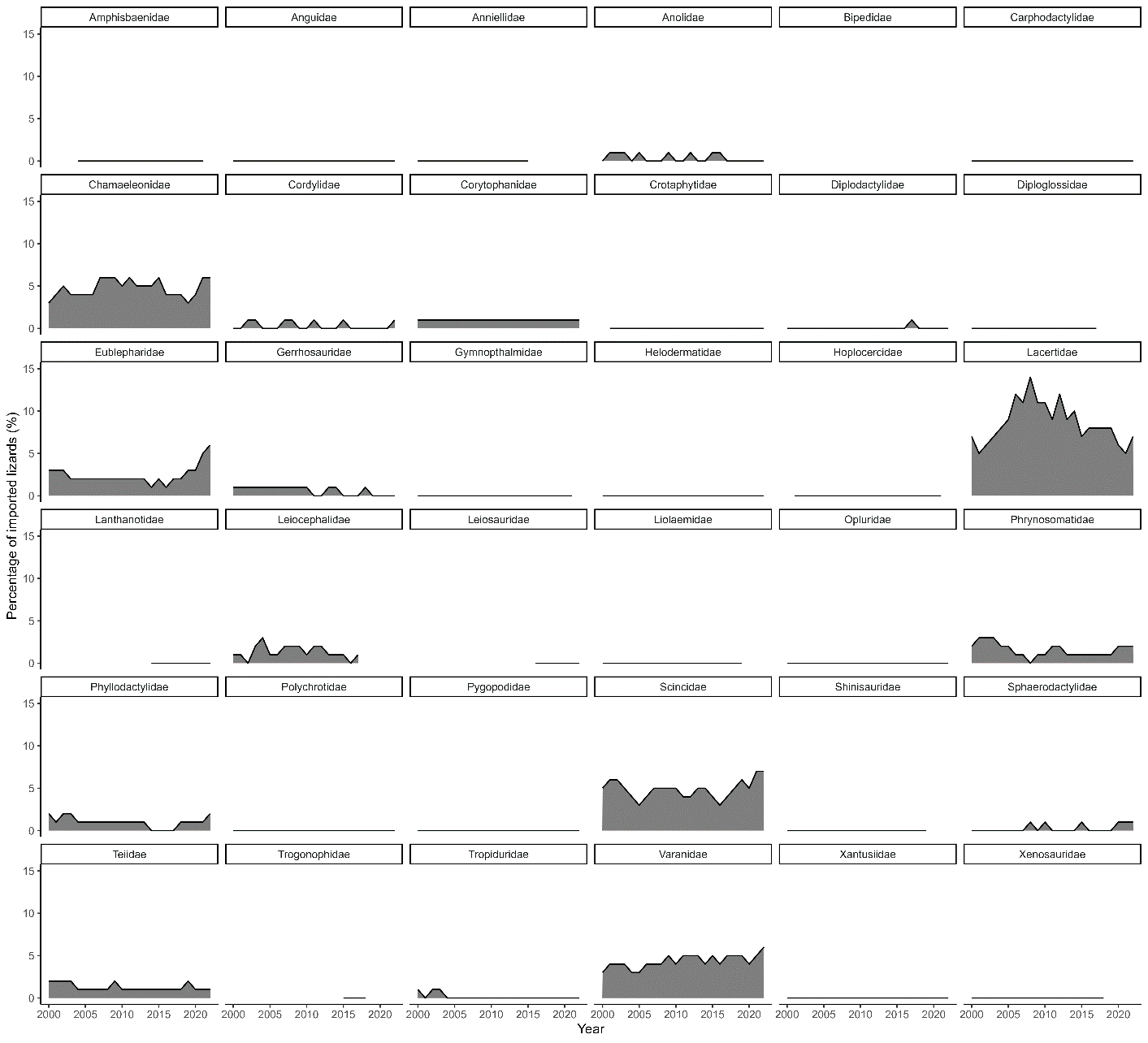


Figure S1. Percentage of imported lizards represented at the family level. The percentage of imported lizards into the United States that represented each family between 2000 and 2022 according to the United States Fish and Wildlife Service’s Law Enforcement Management Information System (LEMIS) dataset. Each family is labelled above each plot.
